# Automated regulatory compliance for AI systems in the security domain: The case of dual-use deployment

**DOI:** 10.12688/openreseurope.23137.1

**Published:** 2026-03-28

**Authors:** Giedre Sabaliauskaite, R. Andrew Paskauskas, Evaldas Bružė, Raminta Matulytė, Tomas Lavišius

**Affiliations:** 1 Faculty of Public Governance and Business, Mykolas Romeris University, Vilnius, 08303, Lithuania; 2 Security Research Laboratory, Mykolas Romeris University, Vilnius, 08303, Lithuania

**Keywords:** Regulatory compliance automation; dual-use technologies; ontology-based requirements specification; security-by-design; privacy-by-design; AI Act; GDPR; NATO cybersecurity framework

## Abstract

The rapid expansion of the European Union’s digital regulatory framework and recent adoption of the Artificial Intelligence Act (AI Act) has introduced complex, overlapping compliance obligations for AI systems, and consequently, their developers and users. These challenges are amplified for dual-use AI systems, which may be developed for civilian markets yet deployed in military contexts, requiring alignment with both EU regulatory instruments and defence-specific frameworks such as NATO policies. Existing approaches to regulatory compliance remain largely manual, fragmented, and difficult to scale, particularly when legal requirements must be translated into actionable, system-level specifications for dual-use contexts.

This paper proposes a novel ontology-based methodology for automated regulatory compliance requirements specification for dual-use AI systems. The methodology systematically integrates legal and technical perspectives by structuring compliance obligations across deployment domains (civilian and defence), system lifecycle phases, and requirement categories, including privacy- and security-related obligations. Implemented as a machine-readable knowledge graph using RDF/Turtle, the approach enables executable compliance modelling, where regulatory obligations are formalised as machine-interpretable entities that can be queried, validated, and deployment-specifically configured through semantic reasoning and SPARQL-based analysis.

The methodology is validated through a detailed case study of an AI-powered espionage detection system, demonstrating how context-aware semantic reasoning and SHACL-based validation can ensure that regulatory requirements are consistently specified and mapped to concrete system components. The proposed framework advances the state of the art by providing a rigorous, extensible foundation for compliance-by-design and automated analysis, thereby reducing compliance risk and supporting responsible AI engineering in complex omni-use regulatory environments.

## 1. Introduction

Artificial intelligence (AI) systems are increasingly embedded in socio-technical infrastructures across sectors such as transport, healthcare, public administration, finance, and defence. As they are becoming more autonomous, adaptive, and scalable, there is an urgent need to establish the harmonised rules for their development, placing on the market, and use.

In June 2024, the European Union (EU) has adopted the Artificial Intelligence Act (AI Act) – the world’s first comprehensive regulatory framework for AI ( European Commission, 2024a). The AI Act forms part of a broader and rapidly expanding digital regulatory ecosystem, that includes General Data Protection Regulation (GDPR), the Data Act, the Digital Services Act (DSA), the Digital Markets Act (DMA), the Cyber Resilience Act (CRA), and the NIS2 Directive among other regulations, aimed at ensuring trust, safety, and competitiveness of Europe’s digital economy ( European Parliament, 2025). Together, these regulations impose complex, overlapping, and evolving compliance obligations on developers, providers, and deployers of AI systems.

The AI Act defines technology-neutral legal requirements related to risk management, data governance, technical documentation, record-keeping, transparency, human oversight, accuracy, robustness, and cybersecurity. In addition, to operationalize their implementation, technical standards are being developed by the CEN-CENELEC JTC 21 (AI), a joint committee by the European Committee for Standardization (CEN) and the European Committee for Electrotechnical Standardization (CENELEC). The Committee is planning to develop circa 35 technical standards to support the AI Act ( Kilian et al., 2025).

The AI standardization in EU faces numerous challenges, such as the overwhelming volume and fragmentation of relevant standards, high compliance costs, and limited participation of start-ups and Small and Medium-sized Enterprises (SMEs) in standardisation processes ( Kilian et al., 2025). AI system developers must navigate the cumulative and sometimes ambiguous interaction between multiple digital laws, where concepts such as risk, accountability, transparency, and security are defined differently across legal instruments. This creates substantial interpretative burdens and increases the risk of inconsistent or incomplete compliance, particularly where requirements must be translated into technical and organisational system specifications.

The growing complexity, dynamism, and interdependence of EU digital regulation call for more systematic, scalable, and adaptive approaches to compliance. Automated regulatory compliance requirements specification, which uses computational methods to identify, structure, formalise, and update regulatory obligations applicable to AI systems, offers a promising pathway to address these challenges. By enabling machine-readable representations of legal and standardisation requirements, automation can support early compliance-by-design, reduce ambiguity, improve traceability between legal norms and technical controls, and lower barriers to compliance for resource-constrained actors, such as SMEs.

The importance of automated compliance specification is further amplified in the context of dual-use AI systems, where systems developed for civilian purposes may also be deployed in defence or security contexts. In this article, dual-use systems are the systems, which can include software and hardware, and which can be used for both civil and military purposes.

Recent scholarship ( Kremidas-Courtney, 2025) suggests that the traditional binary classification of dual-use is increasingly inadequate, advocating instead for an “omni-use by design” philosophy where AI systems operate simultaneously across civilian and security domains. AI systems used for surveillance, target recognition, or decision support often fall at the intersection of civilian AI regulation, sector-specific safety standards, and defence-specific legal frameworks. From an engineering standpoint, this creates “additive” complexity: while military applications may benefit from partial exemptions under the AI Act, dual-use systems often remain subject to a cumulative burden where defence standards (e.g., NIAP, NSP) must layer on top of a civilian GDPR and NIS2 baseline ( Cichocki, 2025; Ndiaye et al., 2026).

This paper investigates how automated approaches to regulatory compliance requirements specification can support compliance with the EU AI Act and the wider EU digital regulatory framework for AI systems. Building on insights from European AI standardisation and the interplay between the AI Act and other digital legislation, the paper argues that automation is not merely a technical optimisation, but a necessary governance mechanism to operationalise abstract legal norms within complex AI systems.

Moreover, this paper proposes a novel ontology-based six-step methodology which provides a systematic approach to specifying regulatory compliance requirements for dual-use AI systems. The methodology systematically integrates legal and technical perspectives by structuring compliance obligations across deployment domains, system lifecycle phases, and requirement categories. Unlike prior ontology-based compliance models limited to single regulatory frameworks, this study introduces a cross-domain executable compliance architecture capable of representing layered EU and defence governance structures within a unified semantic model.

Proposed methodology leverages semantic reasoners and formal constraint languages to address the “interpretative burden” of 2025 regulatory guidelines, providing a structured foundation for real-time compliance automation in high-risk environments like 5G infrastructure and maritime cybersecurity ( Khan et al., 2025; Paskauskas, 2025). Furthermore, a case study on an espionage detection system is included to validate the proposed methodology through the integration of ontology-driven privacy obfuscation and re-identifiability metrics ( Topalli & Badii, 2025).

The remainder of this paper is structured as follows. Section 2 provides the background information on AI systems, including their development lifecycle, regulatory landscape, current practices and related work in the area of automating regulatory compliance. Section 3 presents a novel methodology for dual-use AI system regulatory compliance requirement specification. Further details on how the regulatory compliance requirements are specified are provided in Section 4. A case study is included in Section 5, aimed at validating the proposed methodology. Finally, section 6 summarizes the paper, discusses its major contributions and limitations, identifies directions for future work, and concludes the paper.

## 2. Background and related work

### 2.1. AI system and its development lifecycle

European Commission Guidelines define AI system as a “machine-based system that is designed to operate with varying levels of autonomy and that may exhibit adaptiveness after deployment, and that, for explicit or implicit objectives, infers, from the input it receives, how to generate outputs such as predictions, content, recommendations, or decisions that can influence physical or virtual environments” ( European Commission, 2025a).

This definition comprises seven main elements ( European Commission, 2025a):

1) A *machine-based system*, that includes both the hardware and software components that enable the AI system to function.
2) *Autonomy*: AI system is designed to operate with ‘some degree of independence of actions from human involvement and of capabilities to operate without human intervention’.
3) *Adaptiveness after deployment*: self-learning capabilities, allowing the behaviour of the system to change while in use. The new behaviour of the adapted system may produce different results from the previous system for the same inputs.
4) *Objectives*: AI system is designed to operate according to one or more objectives, that could be explicit or implicit. Explicit objectives refer to clearly stated goals that are directly encoded by the developer into the system. Implicit objectives refer to goals that are not explicitly stated but may be deduced from the behaviour or underlying assumptions of the system. These objectives may arise from the training data or from the interaction of the AI system with its environment.
5) *Output generation*: Ability of a system to derive outputs from given inputs.
6) *Outputs that can influence physical or virtual environments*: The ability of a system, to generate outputs, such as predictions, content, and recommendations, based on inputs it receives and using machine learning and logic and knowledge-based approaches, is fundamental to what AI systems do and what distinguishes those systems from other forms of software.
7) *Interaction with the environment*: AI systems are not passive – they actively impact the environments in which they are deployed. The influence of an AI system may be both to tangible, physical objects (e.g., autonomous vehicle) and to virtual environments, including digital spaces, data flows, and software ecosystems.

Similar to other machine-based systems, the development of an AI system follows a structured lifecycle that spans from initial conception to eventual decommissioning and encompasses multiple distinct stages. AI system development lifecycle, defined by ISO/IEC ISO/IEC 22989 standard ( ISO/IEC, 2022), comprises of several phases, including (1) Inception, (2) Design and development, (3) Verification and validation, (4) Deployment, (5) Operation and monitoring, (6) Re-evaluation, and (7) Retirement. Some of these stages can be repeated throughout lifecycle. E.g., Design and development can be repeated to implement updates and required changes to AI system, followed by Verification and validation and Deployment stages.

The lifecycle stages are often grouped into two main phase – pre-deployment or ‘building’ phase of the system (stages 1–3), and post-deployment or ‘use’ phase (stages 4–7) ( European Commission, 2025a).

AI systems are frequently integrated into broader software ecosystems that follow comparable development lifecycles. These typically encompass the phases of requirements analysis, system design, development and implementation, testing and acceptance, deployment and integration, as well as ongoing maintenance and eventual decommissioning ( ENISA, 2019).

### 2.2. Regulatory landscape

Developing AI systems within the EU requires the integration of legal considerations from the earliest stages of the technology lifecycle. Achieving both operational and regulatory compliance necessitates a comprehensive understanding of EU-level legislation, national legal frameworks, sector-specific requirements, and applicable international standards. This section includes a non-exhaustive overview of the illustrative regulatory frameworks that can affect the development of dual-use AI systems.

At the EU level, for many years, global discussions on AI regulation lacked clear direction. The EU finally addressed this gap in 2024 by adopting the first comprehensive AI regulation globally—the AI Act ( European Commission, 2024a). The Act imposes obligations on AI providers and deployers, categorized by risk level: (1) unacceptable risk; (2) high risk; (3) limited risk; and (4) minimal risk ( European Commission, n.d.). However, its scope excludes AI systems used exclusively for military, defence, or national security purposes (Art. 2(3)). The AI Act foresees a number of requirements that systems and organisations developing or deploying them need to comply with – for example, for high-risk systems, establishing a human in the loop factor, conducting a fundamental rights impact assessment and others.

Another EU regulation, GDPR ( European Commission, 2016a), applies whenever an AI system processes personal data, wholly or partly by automated means (Art. 2(1)), which effectively includes most AI systems. GDPR establishes a comprehensive framework for personal data processing, covering principles such as data minimisation and storage limitation, legal bases, and other operational requirements. For example, under the GDPR, a Data Protection Impact Assessment (DPIA) must be carried out for any new project that is likely to pose a high risk to individuals’ personal data. Similar to the AI Act, GDPR excludes certain processing activities from its scope—for example, processing necessary for common foreign and security policy (Art. 2(2)(b)) or by competent authorities for law enforcement purposes (Art. 2(2)(d)). In the latter case, for law enforcement authorities processing data for law enforcement purposes, the Law Enforcement Directive (LED) applies ( European Commission, 2016b). As related to the personal data processed for national security purposes, member states usually establish their own rules. For example, Lithuania has adopted the Law of the Republic of Lithuania on the Legal Protection of Personal Data Processed for the Purposes of the Prevention, Investigation, Detection or Prosecution of Criminal Offences, the Execution of Sentences or National Security or Defence ( Lietuvos Respublikos Seimas, 2011) which implements the LED as well as sets additional rules in relation to data processing for the purposes of national security and defence.

To strengthen EU cyber resilience, a comprehensive cybersecurity framework has been introduced – the NIS2 Directive. It sets specific management and technical requirements for critical infrastructure organizations (entities) operating in designated sectors ( European Commission, 2022a). Although AI system providers are not always classified as cybersecurity subjects, supply chain obligations may require compliance with NIS2. Additionally, the CRA may apply to certain systems ( European Commission, 2024b). Article 2(1) states that the CRA covers “products with digital elements made available on the market, the intended purpose or reasonably foreseeable use of which includes a direct or indirect logical or physical data connection to a device or network.” However, Article 2(7) exempts products developed or modified exclusively for national security or defence purposes, or those designed to process classified information.

A key EU-level regulation governing technologies with both civilian and defence applications is the Dual Use Regulation ( European Commission, 2021a). It defines “dual-use items” as goods, software, and technology that can serve both civil and military purposes (Art. 2(1)). The Regulation establishes a Union-wide framework for controlling exports, brokering, technical assistance, transit, and transfer of such items.

In addition to EU-level regulations, national legislation can significantly influence AI system development and deployment. For instance, NIS2, as a directive, requires transposition into national law, such as Lithuania’s Cybersecurity Law ( Lietuvos Respublikos Seimas, 2024). Other general laws may also affect compliance. While GDPR provides a comprehensive EU-wide data protection framework, Member States often adopt supplementary national data protection laws introducing additional requirements beyond GDPR.

Depending on the way that AI system is trained and further used, intellectual property considerations may be relevant. OECD, for example, has issued the report on intellectual property issues where AI systems are trained on scraped data ( OECD, 2025). While the report covers many jurisdictions, it emphasises that in relation to the EU, such matters are essentially governed by the Directive on Copyright and Related rights in the Digital Single Market ( European Commission, 2019) and then further to a certain extent repeated in the AI Act.

In addition, sector-specific regulations may apply. For example, the financial sector is mostly exempt from NIS2 as it is governed by the Digital Operational Resilience Act (DORA), the primary cybersecurity framework for financial entities ( European Commission, 2022c). DORA imposes strict cybersecurity requirements on ICT service providers. Consequently, if an AI provider qualifies as an ICT service provider under DORA, its systems must comply with these requirements. Other sectoral regulations—such as those related to healthcare, medical devices, and employment—may similarly impact AI systems.

As the EU lacks exclusive competence in defence regulation, frameworks governing AI systems in this domain are established at the national level. General principles may derive from international public law. Military alliances such as NATO issue guidance and recommendations rather than binding requirements, which are regarded as best practice standards. However, while certain guidelines are important but rather more of a recommended standard in the defence sector, the applicable international humanitarian law (IHL) standards still bind the development and applicability of AI technologies. The IHL legal framework today consists mainly of the four Geneva Conventions of 1949, their additional protocols ( ICRC, 2026a) and customary law ( ICRC, 2026b) and lays down which actions can be taken during the warfare, including when AI is used during armed conflict ( Cuypers, 2023).

Defence deployments do not automatically displace EU legal obligations. Where a system is marketed or made available within the EU, EU regulatory requirements apply to that placement. Where the same system is deployed within NATO infrastructures, additional accreditation and security control requirements arise. The resulting compliance structure is cumulative rather than substitutive: EU market obligations operate alongside defence accreditation requirements, subject to applicable exemptions under EU law.

The International Committee of Red Cross (ICRC) has highlighted the following areas in which AI is being developed for use in warfare, which, among others, can raise significant legal questions: (a) integration in weapon systems, particularly autonomous weapon systems, (b) use in cyber and information operations, (c) underpinning military ‘decision support systems’ ( ICRC, 2023). According to the ICRC, international humanitarian law requires that humans, not machines remain in control of AI used in warfare. While AI may support military operations, human combatants should retain responsibility for the actions made in warfare ( ICRC, 2021). In other words, where AI is used in warfare, its design must, from the beginning, ensure the compliance with IHL obligations.

Finally, the development and deployment of AI, including for military purposes, should always consider the protection of fundamental rights which are primarily established in the European Convention on Human Rights ( European Court of Human Rights, 1950), Charter of Fundamental Rights of the EU ( European Commission, 2012) and national constitutions.

Today, developing software and AI systems requires navigating an increasingly intricate regulatory landscape. Prior to initiating development, it is essential to clearly define: (a) the intended end user, (b) the categories of data the system will process, (c) the system’s primary purpose, and (d) the geographical location in which the system will be developed and deployed.

Based on these parameters, a comprehensive compliance matrix for AI systems can be developed, enabling the identification and resolution of potential regulatory conflicts. For instance, if an AI system is intended for defence use only, many civilian regulatory requirements may not apply. Conversely, for civilian AI systems, the jurisdiction where the AI system is manufactured and (or) placed determines the applicability of specific international, EU-level and national legislation. When personal data is processed, it is essential to assess both the developer and the end-user to determine whether the GDPR, the Law Enforcement Directive or other pieces of data protection legislation apply.

The compliance matrix becomes increasingly complex as additional contextual and operational factors are introduced. It must be noted, therefore, that in the legal scholarship, it is emerging that real applicability of the exemptions included under the certain EU regulations such as the GDPR or AI Act is uncommon in practice, one of the reasons being that governments and militaries often tend to rely on private companies to develop AI. As a result, it is argued that dual-use AI systems may be covered by GDPR/LED (as well as the AI Act for non-military use case scenarios), and only a small part of in-house defence/national security-related processing is purely exempt from the said regulations ( Powell, 2024; Vogiatzoglou, 2024; Juric, 2024).

### 2.3. AI Act risk classification in dual-use contexts

The regulatory classification of AI systems under the AI Act depends primarily on their intended purpose and deployment context ( European Commission, 2024a). For dual-use AI systems, classification requires careful distinction between civilian market placement and defence-only application.

Under Article 6 of the AI Act, an AI system is classified as high-risk where it is either:

(a) intended to be used as a safety component of a product subject to third-party conformity assessment under Union harmonisation legislation, or
(b) listed in Annex III.

Annex III includes, inter alia, AI systems used in:

• biometric identification and categorisation of natural persons,
• management and operation of critical infrastructure,
• law enforcement,
• migration and border control,
• access to essential private and public services.

An espionage detection system deployed in civilian corporate or public-sector environments would require analysis under Annex III, particularly if it performs biometric identification or is used within critical infrastructure contexts. However, a system that detects unauthorised photography attempts without performing identity recognition or categorisation may not automatically fall within the biometric identification category. The classification therefore depends on functional scope rather than technological capability alone.

In any case, it is necessary to assess whether the designed system does not automatically fall under the list of prohibited AI practices under the AI Act. The AI Act prohibits certain practices under Article 5, including specific forms of remote biometric identification in publicly accessible spaces (subject to limited exceptions). Dual-use systems must therefore ensure that civilian variants do not implement prohibited functionality.

Article 2(3) excludes AI systems developed or used exclusively for military, defence, or national security purposes. This exemption is narrow. Where a system is placed on the EU market for civilian use, even if technically capable of defence deployment, the Regulation applies to that civilian deployment. Providers of dual-use AI systems must therefore design compliance mechanisms that accommodate both regulated civilian contexts and defence accreditation environments.

In addition to high-risk classification, providers of high-risk AI systems must comply with obligations including:

• risk management systems (Art. 9),
• data governance measures (Art. 10),
• technical documentation (Art. 11),
• logging capabilities (Art. 12),
• transparency and provision of information to users (Art. 13),
• human oversight (Art. 14),
• accuracy, robustness, and cybersecurity requirements (Art. 15).

These obligations map naturally onto lifecycle phases addressed in the proposed methodology. Risk management and data governance correspond primarily to the design and development phase, while logging, oversight, and monitoring obligations extend into deployment and operational phases.

For dual-use AI systems, the AI Act therefore operates as the civilian compliance baseline. Defence deployments may fall outside the Regulation where exclusively military; however, when personal data is processed, GDPR obligations may continue to apply subject to applicable exemptions. The additive nature of compliance in dual-use systems reinforces the need for structured, lifecycle-based modelling capable of distinguishing domain-specific obligations while preserving traceability.

Companies developing AI systems with potential dual-use applications (e.g., drones, advanced materials software, certain sensors) should consider the AI Act’s compliance requirements from the design phase, particularly if civilian market access is desired ( RAND, 2024). In addition, it may be necessary to clearly separate the military and civilian use cases and ensure that the civilian variants meet all relevant AI Act and GDPR requirements, which may demand separate documentation and conformity assessments.

### 2.4. Current practices in regulatory compliance

Although the above regulations are mandatory, the evolving market and regulatory environment increasingly demands clearer implementation guidance. This section describes recent practices and supporting measures for achieving regulatory compliance.

Technologies subject to the AI Act face several regulatory challenges stemming from ambiguities in the legislation. To support effective implementation, the EU and NATO have introduced policy measures that clarify requirements, refine definitions, and provide guidance essential for developing and deploying relevant technological solutions.

Furthermore, EU regulation, such as CRA, the NIS2 Directive, and the EU AI Act, along with sector-specific safety and cyber security standards, such as IEC 61508 ( IEC, 2010) and ISA/IEC 62443 ( IEC, 2018), use risk-based approaches. However, they differ considerably in scope, language, and application level, resulting in regulatory and technical gaps between horizontal legislation and sector-specific standards ( Ndiaye et al., 2026).

In this study, an additional challenge arises when the technologies under development fall within a dual-use domain, requiring compliance not only for civilian (EU and national level) but also for defence and security (NATO and national level) contexts, where compliance obligations arise through accreditation and governance mechanisms rather than supranational legislation. Within the NATO framework, AI-enabled systems deployed in Alliance environments are subject to security accreditation requirements defined in instruments such as the NATO Information Assurance Policy (NIAP), the NATO Security Policy (NSP), and the NATO Cyber Defence Policy.

These instruments function as binding governance frameworks within the NATO, implemented through procurement conditions, security clearance regimes, certification processes, and operational directives. While not legislative acts comparable to EU regulations, they establish mandatory requirements for systems intended for deployment within NATO environments, including controls related to encryption, access management, incident reporting, secure data exchange, and personnel vetting.

Compliance in defence deployments is, therefore, mainly achieved through accreditation processes and security authorisation rather than conformity assessment under EU market legislation. In a simplified way, for dual-use systems, this creates a layered compliance environment: EU regulatory obligations apply where the system is placed on the EU market or processes personal data within EU jurisdiction, while NATO accreditation requirements apply where the system is deployed within Alliance-controlled infrastructures.

This distinction is important for methodological modelling. The ontology must represent both legally binding EU instruments and defence accreditation frameworks while preserving their different normative statuses.

AI is inherently dual-use, and NATO, therefore, places significant emphasis on its governance through key policy and implementation documents that regulate its application in the defence domain while encouraging collaboration with the civilian sector to support innovation ( NATO’s Strategic Warfare Development Command, n.d.). Several high-level documents outline the core principles and strategic direction guiding NATO’s use of AI.

The NATO AI Strategy, which was adopted in 2021 and revised in 2024, emphasizes the need for reliable implementation of AI in NATO structures, while adhering to key principles: Lawfulness, Responsibility and Accountability, Explainability and Traceability, Reliability, Governability, and Bias Mitigation. This means that any AI technologies implemented in this domain must satisfy accreditation criteria derived from NATO AI governance principles. The strategy identifies that any data adapted to AI must be reliable and free from potential biases. In this sense, the dual-use nature of AI focuses number of challenges, such as “integrating civilian-market solutions into defence and the need to mitigate risks from adversarial use, such as AI-enabled disinformation and information operations that threaten democratic trust” ( NATO, 10 July 2024a).

Therefore, responsible AI certification standards, assessment templates, constant monitoring of technology development trends should be integrated into policy practices. Moreover, the Strategy highlights increased cooperation with the EU as a key objective to facilitate information exchange and the sharing of best practices in AI safety and accreditation. Within the scope of the Strategy, any technology applied in the NATO or dual-use domain, must ensure the application of security-by-design principles, to confirm that AI applications are safe and compliant with international law before deployment.

In addition, Data Strategy for the Alliance ( NATO, 5 May 2025) and NATO’s Digital Transformation Implementation Strategy ( NATO, 17 October 2024b) demonstrate NATO’s strong commitment to becoming a data-driven organization:

• The Data Strategy for the Alliance emphasizes that NATO transitions into a data-centric organization. The strategy clearly highlights security-by-design and privacy-by-design principles through the implementation of a “Data Centric Reference Architecture” and the use of modern standards like STANAG 5636 to ensure semantic interoperability ( NATO, 2022). These are important prerequisites to implement any AI-driven technology across the Alliance. Moreover, the strategy refers to necessity of “adhering to international agreements and approved NATO policies regarding data protection and legal restrictions” ( NATO, 5 May 2025). It should be implemented by secure shared data spaces, which could help to mitigate risks associated with dual-use technologies.
• The NATO’s Digital Transformation Implementation Strategy emphasizes that data-driven decision-making within the Alliance has to possess interoperability across different domains. This requires the commitment to “security and interoperability engineered by design” ( NATO, 17 October 2024b). Thus, relevant standards and coherent governance are needed to ensure that capabilities remain resilient and future-proof. Moreover, the strategy emphasizes the necessity to integrate cybersecurity and cyber defence practices into operational domain, connecting them to NATO and Allied initiatives to reinforce the protection of trusted data and infrastructure. This underscores the shared responsibility of Allies to address data management and protection requirements. Any technology, which has the potential to be disseminated among the members of Alliance, must be fitted to these requirements.

The NATO policy documents referenced above highlight a significant strategic shift in the technological domain—particularly in relation to AI—while also acknowledging the challenges that dual-use technologies must meet in order to comply with both defence-sector requirements and the broader AI and data protection obligations applicable in the civilian domain.

The following are the EU policy practices relevant to dual-use application domain. The Action Plan on Synergies between Civil, Defence and Space Industries ( European Commission, 2021b) emphasizes the use of dual-use technologies like AI for both commercial and military applications to boost EU innovation. The Commission Communication “Roadmap on critical technologies for security and defence” ( European Commission, 2022b) defines strategic framework for the EU to achieve technological sovereignty, reduce dependencies on third countries for technologies, including AI, semiconductors, and cybersecurity. The European Defence Industrial Strategy ( European Parliament, 2024) promotes the integration of civilian innovation (often AI-driven) into the defence sector, further blurring the lines the AI Act seeks to regulate.

In addition, the European Policy Centre report, “From Dual-Use to Omni-Use: Securing Europe’s Future” ( Kremidas-Courtney, 2025), argues that traditional dual-use classifications are no longer relevant in the technological advancement era, because most of the advanced technologies (including AI) operate simultaneously across different domains, like defence, health, industry, creating an “omni-use” approach. The article argues that strict binary logic of dual-use slows innovation and imposes unnecessary compliance burdens. To address this, it recommends a shift toward “omni-use by design” in research and innovation frameworks. This is supported in Cichocki (2025), where the application of AI in maritime cybersecurity both as an offensive weapon and a defensive tool is explored.

Commission Guidelines on Prohibited AI Practices ( European Commission, 2025b) explicitly emphasize that “unacceptable risk” (e.g., remote biometric identification) must be removed from AI-driven technologies, which aim to be disseminated in the EU whether in civil or dual-use domains. The guidelines specifically address practices such as harmful manipulation, social scoring, and real-time remote biometric identification, among others.

Ensuring the consistent, effective, and uniform application of the AI Act across the EU is essential; therefore, the scope for divergent interpretations of its prohibitions should be minimized. The accompanying guidelines provide legal clarification and practical examples to assist stakeholders in understanding and implementing the Act’s requirements. Nevertheless, authoritative interpretations under EU primary law remain exclusively within the competence of the Court of Justice of the European Union (CJEU). Namely, the CJEU cases set the tone for further implementation AI Act and GDPR related practices. Several cases already emerged, showing case law in what ways and to what extent AI technologies can correlate with GDPR requirements, including aspects of national security, data protection, automation, and data anonymization.

In the case “La Quadrature du Net”, CJEU ruled that Member States cannot cite “national security” to issue blanket mandates for mass data retention ( Court of Justice of the European Union, 2020). This is crucial for the AI Act’s dual-use scope: it prevents governments from easily labelling a civilian AI surveillance tool as “national security” just to bypass EU regulations. Moreover, the Court established a critical exception for national security: when a Member State faces a “genuine and present or foreseeable” serious threat to its national security, it may temporarily order the general retention of such data. This preventive measure must be strictly limited in time and subject to effective review by a court or an independent administrative body to prevent abuse.

The following are a few examples of CJEU rulings related to automated decisions:

• In the case “Ligue des droits humains”, CJEU emphasized that automated processing systems must have reliable, non-discriminatory criteria and human review ( Court of Justice of the European Union, 2022). This sets the judicial standard for the “human oversight” or “human in the loop” requirements in the AI Act.
• In “SCHUFA Holding AG”, the Court clarified that any organization providing automated risk-based scores-including those for identity verification or fraud detection-may be subject to the strict requirements of Article 22 ( Court of Justice of the European Union, 2023). Under this provision, such automated decisions are generally prohibited unless they are necessary for a contract, authorized by law.
• Finally, the CJEU ruling in “Dun & Bradstreet Austria GmbH” established that individuals have a right to know the “logic involved” in an automated decision ( Court of Justice of the European Union, 2025). This directly impacts providers of high-risk dual-use AI who might try to hide their algorithms.

In summary, regardless of the domain (whether it is NATO policy and accreditation frameworks, or EU legal regulations), to a certain extent, AI-enabled technologies must balance and not exceed the limits of the AI Act and GDPR requirements, which is essentially confirmed by both policy documents and currently emerging case law.

### 2.5. Related works on automating regulatory compliance

A recent study on requirements engineering for regulatory compliance in software systems ( Kosenkov et al., 2025) identifies numerous persistent challenges, including the abstract nature of regulations, conflicts with existing practices, the need for new expertise, lack of established principles and methods, regulatory complexity, resource demands, enforcement difficulties, evolving system dynamics, overlapping regulatory frameworks, organizational barriers, and regulatory gaps. Out of the 280 publications reviewed in this study, 255 (91%) reported at least one of these challenges. Thus, there is an urgent need for establishing foundational principles and practices that can support development of systematic methodologies and software tools for automating regulatory compliance. Currently, the interpretation and application of regulatory frameworks remain heavily dependent on the specialized knowledge of legal experts ( Elahidoost, 2024).

Generative AI (GenAI) has the potential to enhance regulatory compliance ( Imperial et al., 2025). However, due to above-mentioned challenges – especially, the overlapping regulatory frameworks, regulatory gaps, and conflicts with existing practices – we cannot rely on the AI models yet. Furthermore, questions of liability and accountability persist, particularly regarding responsibility for misinterpretations or incomplete outputs generated by GenAI. Additionally, frequent regulatory updates require continuous model retraining to avoid outdated interpretations. Current standards and regulations are also not fully aligned with GenAI capabilities, and their effective application still demands substantial domain expertise ( Imperial et al., 2025).

The first step toward automating regulatory compliance is the abstraction and formal structuring of legal provisions into a machine-interpretable representation ( Abualhaija et al., 2025). It can be achieved through extracting the concepts – entities, their relationships and attributes – from the legal texts to build a model or ontology. These concepts then serve as the basis for specifying compliance criteria. Once the legal knowledge is appropriately represented, automated methods can support analysts in various compliance related tasks. Abualhaija et al. (2025) identified two possible scenarios for using automated tools for determining regulatory compliance: (i) retrieving compliance relevant information using question answering techniques, and (ii) applying analysis (e.g., Machine Learning) methods to assist in compliance assessment by classifying provided text according to predefined concepts.

An ontology is a formal specification of a conceptualization, defining the relevant concepts and relationships. Ontologies provide explicit meanings for each concept and specify the constraints governing their consistent use. Their reliance on strict logical formalisms makes them more complex to construct as compared to other conceptual models but enables automated validation of knowledge consistency and the use of semantic reasoners to derive new insights. Ontologies are typically expressed in languages that abstract away from data structures and implementation details, making them well suited for integrating heterogeneous databases and supporting interoperability across diverse systems ( Abualhaija et al., 2025).

Thus, Ontology-based Requirements Engineering (ObRE) is a promising way forward for advancing regulatory compliance automation ( Amaral et al., 2021; Guizzardi et al., 2023). The following are several examples of using ontological frameworks to automate regulatory compliance:

• Hosseini et al. (2025) proposed an ontological framework to support security-by-design of industrial control systems by conforming with the IEC 62443 standard.
• Hernandez et al. (2024) introduced a Trustworthy AI Requirements (TAIR) ontology, which provides the basis for mapping the concepts and requirements from the AI Act.
• Guizzardi et al. (2023) proposed an ontological approach for specifying so called “ethicality requirements” – requirements for developing ethical systems, including AI systems.
• Paskauskas (2025) developed an ontology for 5G network security ensuring compliance with EU cybersecurity requirements.
• Orlando & Santoro (2025) proposed a framework that integrates natural language processing (NLP) with semantic and topic modelling techniques for automated analysis of GDPR enforcement decisions.

The major limitations of currently available ontological frameworks for supporting regulatory compliance include:

a) Limited scope: not taking into consideration multiple standards and regulations.
b) Insufficient for dual-use: not addressing the needs of developing dual-use or omni-use technologies.

## 3. Methodology for dual-use AI system regulatory compliance requirement specification

### 3.1. Methodology steps

**Figure 1.**
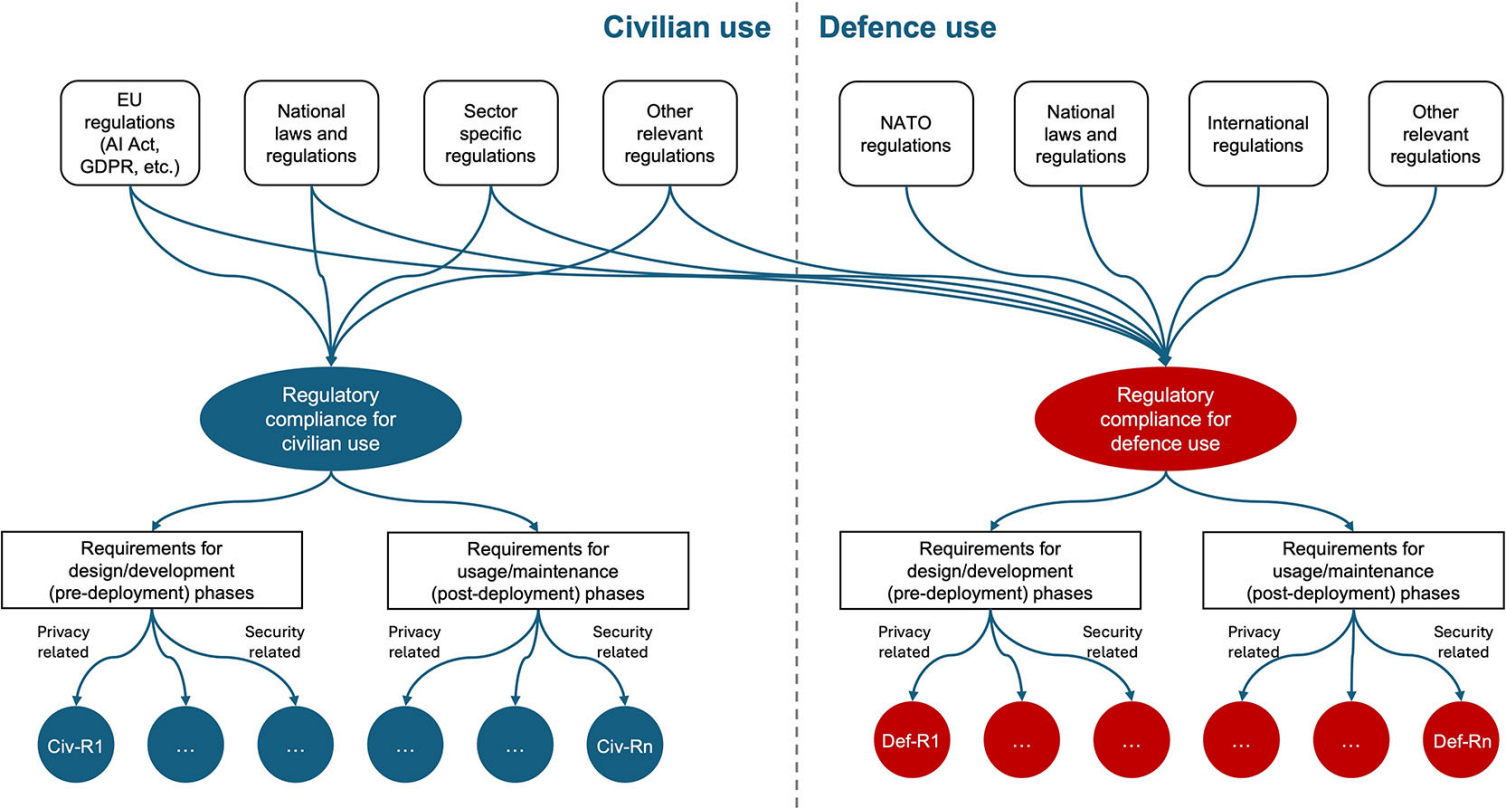
Methodology scope. (Methodology uses civilian and defence regulations as an input to perform regulatory compliance analysis for civilian and defence use respectively and specifies requirements for pre-deployment and post-deployment phases. Requirements can be classified by their type, e.g., privacy-related, security-related).

This section describes the steps of the proposed methodology for ontology-based regulatory compliance requirements specification for dual-use AI systems. Its scope is presented in Figure 1. It used regulations (both, for civilian and defence use) as an input to perform regulatory compliance analysis and specifies requirements for pre-deployment and post-deployment phases. A list of regulation, shown in Figure 1, is only an example and does not include a complete list of regulations relevant for dual-use AI systems.

Methodology comprises six steps, progressing from problem definition through validated knowledge graph deployment.

***Step 1: Problem Framing and Regulatory Scoping***

The first step establishes the dual-use context and identifies the applicable regulatory landscape across both civilian and defence domains.

*Activities:*

• Define the technology under consideration and its potential deployment contexts (civilian, defence, or both);
• Identify applicable civilian regulations (e.g., GDPR, NIS2 Directive, EU AI Act, Cyber Resilience Act, national cybersecurity laws);
• Identify applicable defence policy and accreditation frameworks (e.g., NATO Information Assurance Policy, NATO Security Policy, NATO Cyber Defence Policy, TEMPEST standards, STANAG agreements);
• Document the dual-use compliance challenge: where requirements overlap, diverge, or potentially conflict.

*Outputs:*

• Regulatory scope document listing all applicable frameworks by domain;
• Initial mapping of regulatory hierarchies (international → EU/NATO → national);
• Identification of cross-domain requirements (e.g., GDPR may still apply where personal data processing falls outside national security/defence derogations).

*Rationale:*

Dual-use technologies must satisfy regulatory requirements from fundamentally different legal regimes. Early scoping prevents gaps in compliance coverage and identifies potential conflicts that must be resolved in system design.

***Step 2: Conceptual Framework Development***

The second step translates the regulatory scoping into an explicit conceptual model that structures requirements across domains, lifecycle phases, and categories.

*Activities:*

• Create a visual representation of the regulatory compliance framework showing:
  o Two top-level compliance domains (civilian use, defence use);
  o Regulatory source hierarchies feeding into each domain;
  o Lifecycle phase decomposition (design/development vs. usage/maintenance);
  o Requirement categorization (privacy-related vs. security-related).
• Map design principles to lifecycle phases:
  o Privacy-by-design (GDPR Article 25) → primarily governs design/development phase;
  o Security-by-design (GDPR Article 32) → primarily governs usage/maintenance phase;
• Define the structural parallelism: both domains follow identical phase and category organization to enable systematic comparison.

*Outputs:*

• Conceptual diagram showing dual-domain regulatory framework (as shown in Figure 1);
• Design principle mapping to lifecycle phases;
• Structural template for requirement specification (domain × phase × category).

*Rationale:*

A visual conceptual model serves as a shared reference between legal experts, ontology engineers, and system developers. The structural parallelism ensures that civilian and defence requirements can be systematically compared and that gaps in either domain are immediately visible.

***Step 3: Ontology Design and Implementation***

The third step formalizes the conceptual model as a machine-readable ontology expressed in RDF/Turtle.

*Activities:*

• Define namespace and prefixes (e.g., rc: < http://l3ce.lt/ontology/regulatory-compliance >)
• Create class hierarchy:
  o Root class: RegulatoryCompliance;
  o Domain classes: RegComplyforCivilUse, RegComplyforDefenceUse;
  o Regulatory framework classes: EURegulations, NATORegulations, NatLawsAndRegs;
  o Specific regulations: GDPR, NIS2Directive, NATOSecurityPolicy, etc.;
  o Structural classes: LifecyclePhase, DesignPrinciple, RequirementCategory, Requirement.
• Define object properties:
  o hasPhase — links compliance domain to lifecycle phases;
  o hasRequirement — links phases to specific requirements;
  o derivedFrom — links requirements to source regulations;
  o hasRequirementCategory — classifies requirements as privacy-related or security-related;
  o appliesToDomain — tags requirements for civilian or defence context;
  o hasDefenceEquivalent/hasCivilianEquivalent — maps cross-domain relationships.
• Define datatype properties:
  o requirementIdentifier — unique identifier string (e.g., “CIV-DESIGN-R1”);
  o articleReference — citation to specific regulatory articles;
  o legalBasis — substantive description of the compliance obligation.

*Outputs:*

• RDF/Turtle ontology file defining classes, properties, and structural relationships;
• Documented namespace and prefix conventions;
• Class hierarchy diagram.

*Rationale:*

**Figure 2.**
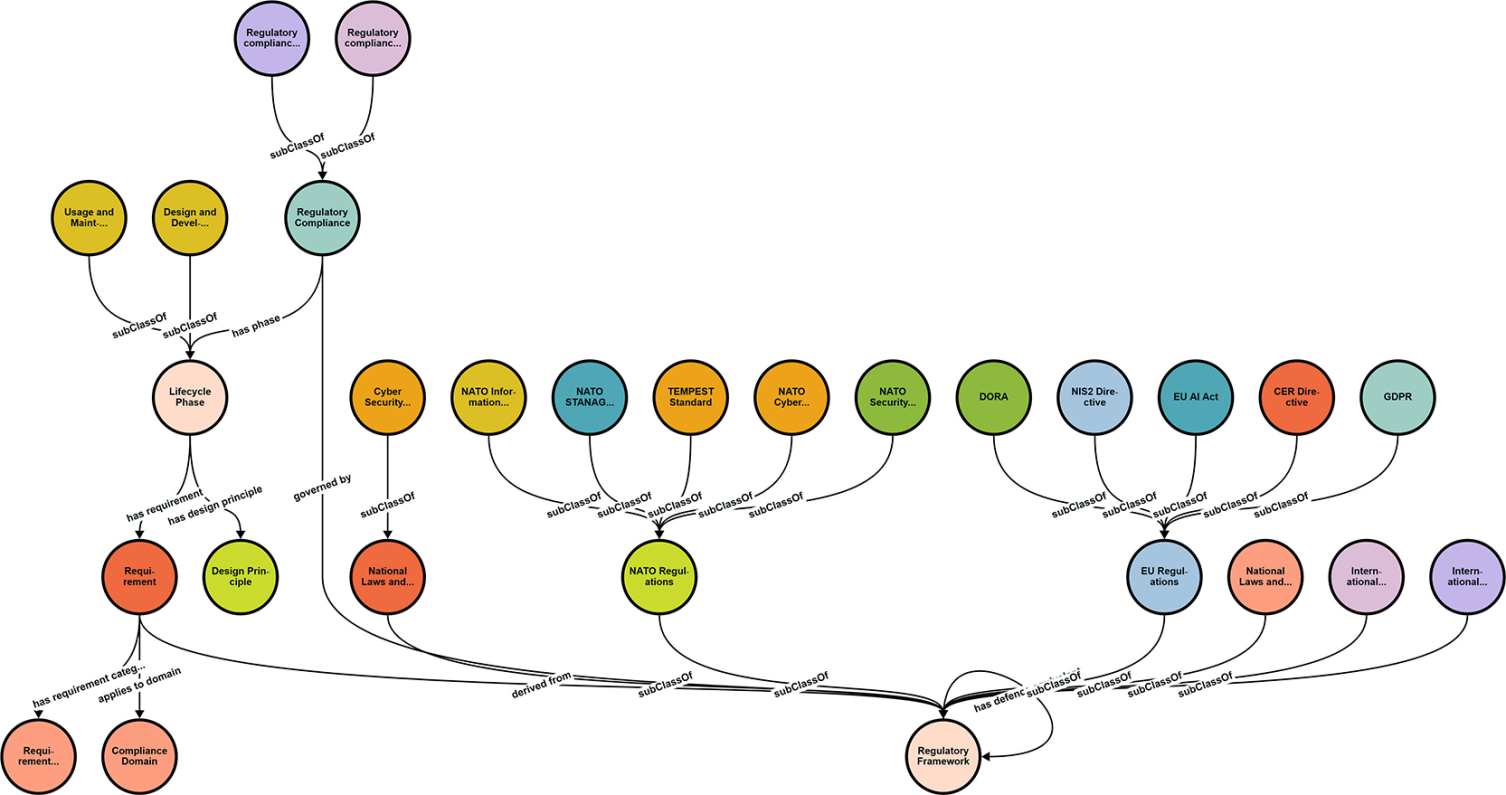
Regulatory compliance ontological framework for dual-use technologies.

RDF/Turtle provides a standardized, interoperable format that can be loaded into knowledge graph platforms (e.g., Stardog, GraphDB) and queried using SPARQL. The formal semantics enable automated consistency checking and reasoning. The visual representation is depicted in the STARDOG Knowledge Graph in Figure 2.

***Step 4: Domain-Specific Requirements Population***

The fourth step instantiates concrete regulatory requirements within the ontological framework, populating both civilian and defence modules.

*Activities:*

• For each domain (civilian, defence), we create requirement instances following the structural template:
  o Design/Development phase: one privacy-related requirement (R1), one security-related requirement (R2);
  o Usage/Maintenance phase: one privacy-related requirement (R3), one security-related requirement (R4);
• Populate each requirement instance with:
  o requirementIdentifier: structured ID reflecting domain, phase, and sequence (e.g., “DEF-USAGE-R4”);
  o rdfs:label: human-readable requirement name;
  o derivedFrom: link to source regulation(s);
  o articleReference: specific article/section citations;
  o legalBasis: substantive text describing the compliance obligation;
  o hasRequirementCategory: classification as PrivacyRelated or SecurityRelated;
  o appliesToDomain: explicit domain tag (CivilianDomain or DefenceDomain);
• Where regulations support decomposition, we create sub-requirements linked to parent (e.g., DEF-USAGE-R4 → DEF-USAGE-R4-ART3, DEF-USAGE-R4-ART5, DEF-USAGE-R4-ART7).

*Outputs:*

• Populated civilian requirements module (Civ-R1 through Civ-R4);
• Populated defence requirements module (Def-R1 through Def-R4);
• Sub-requirements where applicable;
• Complete RDF/Turtle file ready for knowledge graph deployment.

*Rationale:*

The parallel structure across domains (R1–R4 in both civilian and defence) enables systematic comparison and gap analysis. Explicit domain tagging via appliesToDomain provides a reliable partitioning mechanism that supplements class-based inference.

***Step 5: Knowledge Graph Deployment***

The fifth step loads the ontology into a triplestore platform and verifies structural integrity.

*Activities:*

• Create database instance in target platform (e.g., Stardog Cloud);
• Load RDF/Turtle ontology file;
• Verify successful loading by checking triple count;
• Configure visualization settings for knowledge graph exploration;
• Test basic navigation: browse class hierarchy, inspect individual requirement instances.

*Outputs:*

• Deployed knowledge graph accessible for querying;
• Visual representation of regulatory compliance framework;
• Confirmation of structural integrity (all expected classes, properties, and instances present).

*Rationale:*

Knowledge graph deployment enables interactive exploration of the regulatory framework by stakeholders with varying technical backgrounds. Visual navigation supports validation with domain experts (legal, compliance, engineering) who may not be familiar with SPARQL.

***Step 6: SPARQL-Based Validation and Cross-Domain Analysis***

The sixth step validates the framework through structured queries and demonstrates its analytical capabilities for dual-use compliance planning.

*Activities:*

• Design validation queries to verify:
  o All requirement instances are correctly typed and populated;
  o Domain tagging is consistent (appliesToDomain values);
  o Lifecycle phase associations are complete;
  o Regulatory source linkages (derivedFrom) are present.
• Execute cross-domain comparison queries:
  o Retrieve all requirements grouped by domain;
  o Pair civilian and defence requirements by structural position (e.g., CIV-DESIGN-R1 ↔ DEF-DESIGN-R1);
  o Identify shared regulatory sources;
  o Identify domain-specific sources.
• Iteratively refine ontology based on query results:
  o Correct missing or malformed property values;
  o Add missing cross-references;
  o Enhance requirement decomposition where needed.

*Outputs:*

• Validated query results demonstrating framework completeness;
• Cross-domain comparison tables (see Tables 1– 4);
• Documentation of any refinements made during validation;
• SPARQL query library for reuse in future compliance assessments (viz. GitHub repo).

**Table 1.** Summary of six steps of the methodology.

Step	Focus	Key Output
1	Problem Framing & Regulatory Scoping	Regulatory scope document
2	Conceptual Framework Development	Visual model and structural template
3	Ontology Design & Implementation	RDF/Turtle ontology file
4	Domain-Specific Requirements Population	Populated civilian and defence modules
5	Knowledge Graph Deployment	Deployed, navigable knowledge graph
6	SPARQL-Based Validation & Analysis	Validated results and query library

*Rationale:*

• SPARQL-based validation ensures that the ontological framework is complete, consistent, and queryable. The cross-domain comparison queries directly support dual-use compliance planning by revealing where civilian and defence requirements align (shared obligations) and where they diverge (domain-specific security frameworks), as identified in Step 1.

The following are two example queries: A – retrieves all requirements across civilian and defence domains; B – pairs civilian and defence requirements side-by-side. More details on query outputs are provided in Section 4.

*Query A: Retrieve all requirements across both domains*

PREFIX rc: <http://l3ce.lt/ontology/regulatory-compliance>
PREFIX rdfs: <http://www.w3.org/2000/01/rdf-schema>
SELECT ?identifier ?label ?domain ?category ?article ?legalBasis
WHERE {
    ?req a rc:Requirement;
      rdfs:label? label;
      rc:requirementIdentifier? identifier;
      rc:hasRequirementCategory? category;
      rc:appliesToDomain? domain.
    OPTIONAL {?req rc:articleReference? article}
    OPTIONAL {?req rc:legalBasis? legalBasis}
    FILTER(?domain IN (rc: CivilianDomain, rc: DefenceDomain))
}
ORDER BY? domain? identifier



*Query B: Side-by-side cross-domain pairing*

PREFIX rc: <http://l3ce.lt/ontology/regulatory-compliance>
PREFIX rdfs: <http://www.w3.org/2000/01/rdf-schema>
SELECT? civId? civLabel? civArticle? defId? defLabel? defArticle
WHERE {
    ?civReq a rc: Requirement;
        rc:requirementIdentifier? civId;
        rdfs:label? civLabel;
        rc:appliesToDomain rc: CivilianDomain.
    OPTIONAL {?civReq rc:articleReference? civArticle}
    ?defReq a rc: Requirement;
        rc:requirementIdentifier? defId;
        rdfs:label? defLabel;
        rc:appliesToDomain rc: DefenceDomain.
    OPTIONAL {?defReq rc:articleReference? defArticle}
    FILTER (REPLACE(?civId, “CIV”, “”) = REPLACE(?defId, “DEF”, “”)).
}
ORDER BY ?civId



### 3.2. Methodology summary

The six-step methodology provides a systematic approach to specifying regulatory compliance requirements for dual-use AI systems. The focus and key outputs of each step are summarized in Table 1.

The methodology addresses the limitations identified in Section 2.5 by: (a) explicitly considering multiple regulations across both civilian and defence domains, and (b) providing a structured approach for dual-use technology compliance that maintains traceability between legal requirements and technical specifications.

### 3.3. Methodology innovation: from static mapping to executable compliance logic

Existing regulatory compliance approaches in AI governance predominantly rely on static documentation, checklist-based conformity assessments, or manual legal interpretation. Even ontology-based models frequently function as conceptual mapping tools without executable validation capacity.

The methodological contribution of this study lies in transforming regulatory requirements into executable compliance logic. The framework operationalises legal obligations as structured RDF entities linked to lifecycle phases, deployment domains, and regulatory sources, enabling:

1) Query-based compliance verification - SPARQL queries retrieve and compare applicable obligations dynamically depending on deployment context.
2) Cross-domain alignment logic - Structural pairing of civilian and defence requirements supports automated gap analysis.
3) Hierarchical decomposition with traceability - Article-level sub-requirements enable fine-grained mapping between system components and specific legal provisions.
4) Conditional applicability modelling - Domain tagging and potential restriction flags allow the representation of context-dependent regulatory scope (e.g., GDPR scope exemptions and Article 23 restrictions, AI Act military exemption).

This shifts compliance engineering from static documentation toward an executable knowledge representation capable of supporting iterative system design, accreditation preparation, and deployment-specific compliance configuration.

## 4. Specification of regulatory compliance requirements using proposed methodology

This section demonstrates how the methodology works and what results it generates. Currently, ontology captures a subset of applicable regulations (GDPR, NIS2, NATO policies) and their selected features to show how the methodology works. In the future, it should be expanded to include all relevant provisions of the selected regulations as well as the EU AI Act risk classification, Cyber Resilience Act, sector-specific and other relevant regulations and standards.

Tables 2 through 5 present the SPARQL query results showing regulatory compliance requirements across both civilian and defence deployment contexts, organized by domain, lifecycle phase, and requirement category. The results shown in Tables 2– 5 are illustrative examples and do not constitute a complete set of requirements.

**Table 2.** Civilian domain requirements and legal basis.

Req. ID	Requirement	Category	Article Reference
CIV-DESIGN-R1	DPIA and Privacy by Design	Privacy-related	GDPR Article 25 (Data protection by design), Article 35 (DPIA)
*Legal basis*	Data Protection Impact Assessment required before deployment. Data minimization principles must govern image capture design: lens detection must avoid unnecessary recording, capture only what is essential for security verification. Retention periods must be defined during design phase, not post-deployment. System architecture must embed privacy safeguards from inception.		
CIV-DESIGN-R2	Security Architecture by Design	Security-related	GDPR Article 32 (Security of processing), NIS2 Article 21 (Cybersecurity risk-management measures)
*Legal basis*	Encryption architecture must be designed into system from inception. Secure communication protocols required for data transmission to remote monitoring station. Hardened firmware and secure boot mechanisms must be specified during development. Access control frameworks must be architected before deployment.		
CIV-USAGE-R3	Breach Notification and Data Subject Rights	Privacy-related	GDPR Article 33 (Notification to supervisory authority), Article 34 (Communication to data subject)
*Legal basis*	Personal data breaches must be reported to supervisory authority within 72 hours of becoming aware. High-risk breaches require communication to affected data subjects without undue delay. Organizations must implement data subject rights: right of access (Art. 15), right to erasure (Art. 17), right to restriction (Art. 18). Transparency notices required in deployment locations informing individuals of monitoring.		
CIV-USAGE-R4	Operational Cybersecurity Measures	Security-related	NIS2 Article 21 (Cybersecurity risk-management measures), GDPR Article 32
*Legal basis*	Encrypted data transmission required for all communications to remote monitoring server. Access controls must restrict image viewing to authorized personnel only. Comprehensive audit logging of all system access and data processing activities. Incident response procedures must be documented and tested. Vulnerability management and patch deployment processes required. Regular security assessments and penetration testing.		

### 4.1. Regulatory compliance requirements for civilian use

Table 2 shows non-exhaustive examples of civilian domain requirements derived from EU regulations (GDPR, NIS2 Directive), organised by lifecycle phase (DESIGN = design/development; USAGE = usage/maintenance) and category (privacy-related, security-related). Legal basis for each requirement is included in Table 2 as well. It captures the substantive compliance obligations derived from regulatory frameworks; however, the obligations in the following table are included for exemplary purposes only and should be expanded to capture more detailed obligations in real-life scenarios. This enables automated compliance checking and traceability between system design decisions and regulatory mandates.

### 4.2. Regulatory compliance requirements for defence use

Table 3 presents the ontological representation of regulatory compliance requirements for defence-use deployments, organized by lifecycle phase and requirement category. It provides the substantive compliance content for each requirement, showing how the ontological model captures actionable regulatory obligations. Regulatory requirements are assigned to specific lifecycle phases (pre-deployment vs. post-deployment) and categories (privacy-related vs. security-related). In the provided example, requirements are derived from national regulations and NATO frameworks (NIAP, NSP, NATO Cyber Defence Policy), reflecting the dual-use nature of the system under consideration.

**Table 3.** Defence domain requirements and legal basis.

Req. ID	Requirement	Category	Phase	Regulatory Source	Article Reference
DEF-DESIGN-R1	DPIA and Privacy by Design	Privacy-related	Design & Development	Law of the Republic of Lithuania on the Legal Protection of Personal Data Processed for the Purposes of the Prevention, Investigation, Detection or Prosecution of Criminal Offences, the Execution of Sentences or National Security or Defence ^*^	Article 25 Article 18
Legal basis	Data Protection Impact Assessments identify risks, evaluate safeguards, and ensure compliance before systems go live. Embedded in privacy by design and by default principle.				
DEF-DESIGN-R2	NATO Security Controls	Security-related	Design & Development	NIAP, NSP	—
Legal basis	NIAP: Multi-layered security controls including encryption, access controls, and incident response. NSP: Access controls, physical security, & personnel training for classified info.				
DEF-USAGE-R4	NATO Cyber Defence Compliance	Security-related	Usage & Maintenance	NATO Cyber Defence Policy	—
Legal basis	Protection of digital assets from cyber threats, including unauthorised data capture through non-networked means such as cameras.				

* Applicable only in Lithuania and might vary in other Member States.

The SPARQL code looks like this:

PREFIX rc: <http://l3ce.lt/ontology/regulatory-compliance>
PREFIX rdfs: <http://www.w3.org/2000/01/rdf-schema>
PREFIX skos: <http://www.w3.org/2004/02/skos/core>
SELECT? identifier? label? domain? category? article? legalBasis
WHERE {
    ?req a rc: Requirement;
      rdfs:label? label;
      rc:requirementIdentifier? identifier;
      rc:hasRequirementCategory? category;
      rc:appliesToDomain? domain.
    OPTIONAL {?req rc:articleReference? article}
    OPTIONAL {?req rc:legalBasis? legalBasis}
    FILTER(?domain IN (rc: CivilianDomain, rc: DefenceDomain))
}
ORDER BY? domain? identifier



It must be noted, however, that identification of certain requirements may differ depending on the particular system to which the ontological framework is applied. As briefly indicated previously, GDPR includes certain exemptions, including for defence data processing operations. Therefore, for example, the DPIA requirement may not be attributed to defence domain if the system is intended to be used solely by military for military operations which fall outside the EU competences scope. However, as included in. the table, certain data protection obligations as related to defence domain can be established in national laws – for example, the Law of the Republic of Lithuania on the Legal Protection of Personal Data Processed for the Purposes of the Prevention, Investigation, Detection or Prosecution of Criminal Offences, the Execution of Sentences or National Security or Defence, includes the obligation to perform a DPIA (Article 25). When the competent authorities process personal data for the purposes of national security or defence, the Law applies unless otherwise provided for in other laws. In other words, while the GDPR may exclude military operations from its scope, similar obligations still may be required under the national military-related laws. And vice versa – not all data processing operations performed by defence organisations necessarily fall outside the EU regulation, e.g., if defence organisations process data not for exclusively military purposes. Therefore, even regulatory domain distinctions must be done with extreme cautiousness to capture all possible scenarios so that no AI system component slips through the regulatory gaps.

The ontological framework supports hierarchical requirement decomposition, as demonstrated by the granular specification of NATO Cyber Defence Policy obligations, and can be evoked by executing the code below:

PREFIX rc: <http://l3ce.lt/ontology/regulatory-compliance>
PREFIX rdfs: <http://www.w3.org/2000/01/rdf-schema>
SELECT? identifier? label? category? article? legalBasis
WHERE {
    ?req a rc: Requirement;
      rdfs:label? label;
      rc:requirementIdentifier? identifier;
      rc:hasRequirementCategory? category.
    OPTIONAL {?req rc:articleReference? article}
    OPTIONAL {?req rc:legalBasis? legalBasis}
    FILTER (CONTAINS(?identifier, “DEF-”))
}
ORDER BY? identifier



Table 4 illustrates the framework’s capacity for requirement decomposition. The parent requirement DEF-USAGE-R4 (NATO Cyber Defence Compliance) defined in Table 3 is refined into article-level sub-requirements, enabling precise traceability between system components and specific regulatory provisions.

**Table 4.** Example of refining requirements into sub-requirements.

Req. ID	Sub-Requirement	Article Reference	Legal Basis
DEF-USAGE-R4-ART3	Detection Mechanisms	Article 3, Paragraph 2	Entities must deploy defence mechanisms that include the detection and reporting of unauthorised data capture.
DEF-USAGE-R4-ART5	Secure Incident Reporting	Article 5, Paragraph 8	Detected incidents must be reported through secure, encrypted channels.
DEF-USAGE-R4-ART7	Threat Intelligence Sharing	Article 7, Paragraph 6	Threat intelligence must be shared across NATO entities.

### 4.3. Aligning civilian and defence requirements

Table 5 presents direct SPARQL query output demonstrating the framework’s ability to programmatically align civilian and defence requirements by structural position. It includes results from a SPARQL query that automatically pairs civilian and defence requirements by matching their structural position (e.g., CIV-DESIGN-R1 pairs with DEF-DESIGN-R1). This demonstrates the framework’s support for automated cross-domain compliance analysis.

**Table 5.** Aligning civilian and defence domain requirements.

Civilian ID	Civilian Requirement	Civilian Article Reference	Defence ID	Defence Requirement	Defence Article Reference
CIV-DESIGN-R1	DPIA and Privacy by Design	GDPR Article 25, Article 35	DEF-DESIGN-R1	DPIA and Privacy by Design	Law of the Republic of Lithuania on the Legal Protection of Personal Data Processed for the Purposes of the Prevention, Investigation, Detection or Prosecution of Criminal Offences, the Execution of Sentences or National Security or Defence, Article 25, Article 18
CIV-DESIGN-R2	Security Architecture by Design	GDPR Article 32, NIS2 Article 21	DEF-DESIGN-R2	NATO Security Controls	—
CIV-USAGE-R3	Breach Notification and Data Subject Rights	GDPR Article 33, Article 34			
CIV-USAGE-R4	Operational Cybersecurity Measures	NIS2 Article 21, GDPR Article 32	DEF-USAGE-R4	NATO Cyber Defence Compliance	—

The SPARQL query looks like this:

PREFIX rc: <http://l3ce.lt/ontology/regulatory-compliance>
PREFIX rdfs: <http://www.w3.org/2000/01/rdf-schema>
SELECT? civId? civLabel? civArticle? defId? defLabel? defArticle
WHERE {
    ?civReq a rc: Requirement;
        rc:requirementIdentifier? civId;
        rdfs:label? civLabel;
        rc:appliesToDomain rc: CivilianDomain.
    OPTIONAL {?civReq rc:articleReference? civArticle}
    ?defReq a rc: Requirement;
        rc:requirementIdentifier? defId;
        rdfs:label? defLabel;
        rc:appliesToDomain rc: DefenceDomain.
    OPTIONAL {?defReq rc:articleReference? defArticle}
    FILTER (REPLACE(?civId, “CIV”, “”) = REPLACE(?defId, “DEF”, “”))
}
ORDER BY? civId



### 4.4. Regulatory compliance requirement specification summary

In summary, The SPARQL query results, presented in Tables 2- 5, demonstrate three key capabilities of the ontological framework:

1) *Lifecycle-phase organization:* Requirements are systematically allocated to either the design/development phase (where privacy-by-design and security-by-design principles are embedded) or the usage/maintenance phase (where operational compliance obligations apply).
2) *Dual regulatory coverage*: The framework simultaneously captures EU civilian regulations (GDPR) and NATO defence-specific frameworks (NIAP, NSP, Cyber Defence Policy), reflecting the dual-use nature of the system/technology.
3) *Hierarchical decomposition*: High-level requirements can be decomposed into granular, article-specific sub-requirements, supporting both strategic compliance planning and detailed technical validation.

## 5. Case study

This section validates the proposed ontology-based methodology by applying it to a sample dual-use system – espionage detection system, which uses AI technology designed to detect and report unauthorised photographing of classified or confidential information displayed on screens. The system does not perform biometric identification, categorisation, or profiling of individuals; it detects behavioural indicators (e.g., camera lenses directed toward protected displays) without associating detected persons with identifiable biometric templates.

### 5.1. System description

**Figure 3.**
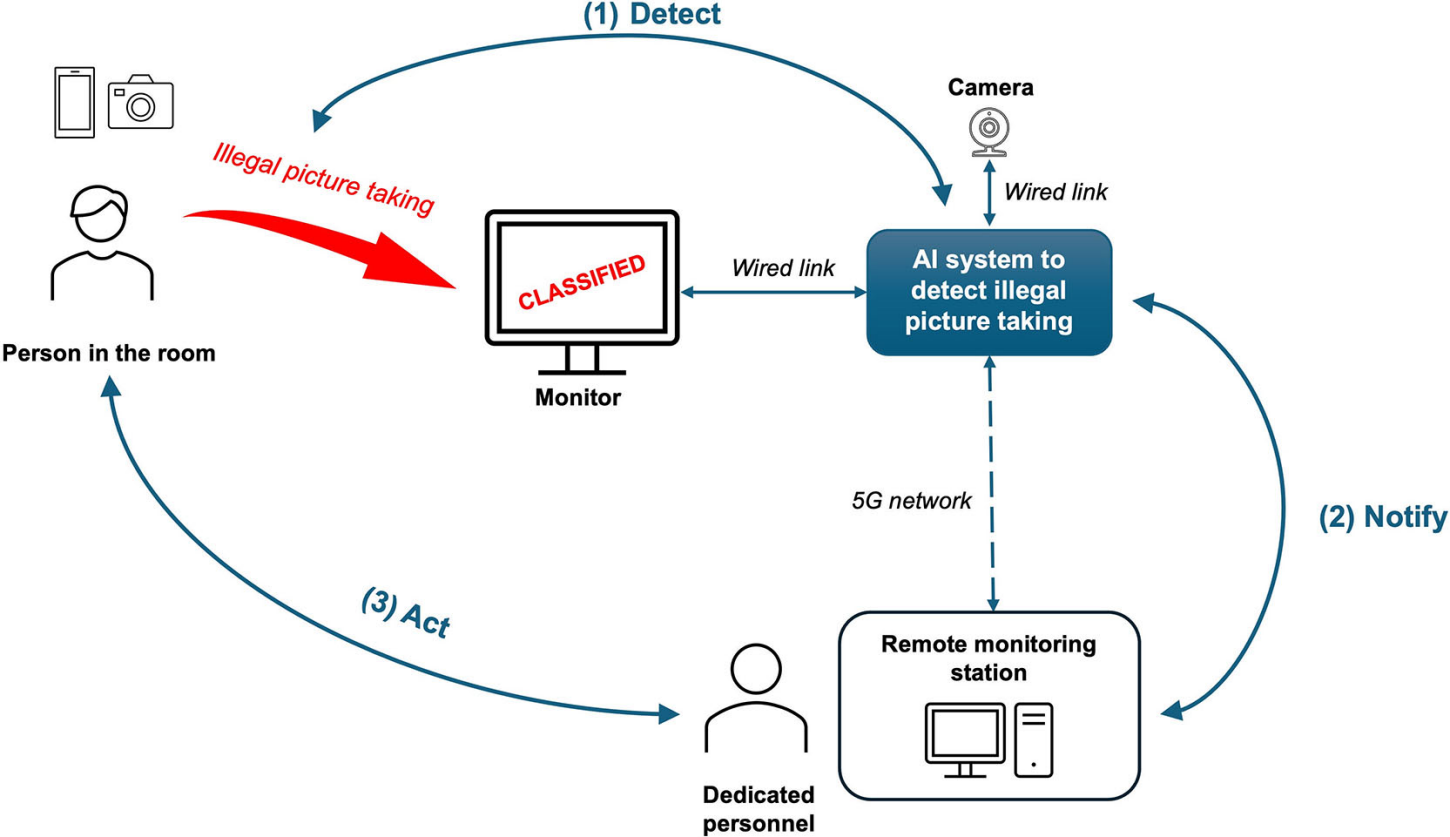
Espionage detection system architecture and operational flow.

The espionage detection system comprises several main components (see Figure 3). It includes: a monitor, which displays sensitive/classified information; an AI system, connected to monitor via Ethernet, which includes software and hardware components to monitor analyse the behaviour of people in the room; a camera connected to AI system via USB for visual monitoring of the room; a remote monitoring station, which communicates with the AI system via 5G network. Beyond basic hardware, the system’s “intelligence” is grounded in an ontology-driven context-aware framework. While the camera and AI module identify unauthorised photography, recent 2025 research suggests that semantic reasoning is required to distinguish between legitimate mobile use and an actual “espionage” event. By integrating a Re-Identifiability Index (RII), the system can mitigate identity leakage during visual monitoring, ensuring that only the “threat” is processed while preserving bystander privacy through adaptive obfuscation ( Topalli & Badii, 2025). Furthermore, AI system could implement additional features such as scene interpretation ( Gómez-Romero et al., 2011) and deepfake and biometric security ( Pn et al., 2024).

Operational workflow of espionage detection consists of three phases (see Figure 3):

(1) *Detect.* Camera monitors the room, where classified information is displayed on a screen. The AI system analyses the video feed to detect attempts at unauthorised picture-taking, such as the presence of camera lenses or smartphone screens pointed at the display.
(2) *Notify.* Upon detection of a potential security breach, the system transmits an alert notification to the remote monitoring station. Notification includes captured image (potentially containing faces of individuals present), AI system’s confidence level, timestamp, information about what was displayed on screen at the time of detection, and monitor’s location data (remote monitoring station could be receiving information from multiple espionage detection AI systems at the same time).
(3) *Act.* Dedicated security personnel at the remote monitoring station receive the alert and take appropriate response actions.

The system is intended for deployment in both civilian contexts (corporate boardrooms, government offices, financial institutions, healthcare facilities) and defence contexts (NATO command centres, military briefing rooms, classified research facilities). This dual-use nature creates overlapping and sometimes divergent compliance obligations that the ontological framework must address.

### 5.2. Component-to-requirement mapping

To validate the ontological framework, we systematically map each espionage detection system component and data flow to the applicable regulatory requirements across both civilian and defence domains. The results are shown in Table 6, which demonstrates how the ontological framework enables systematic mapping of system components to applicable regulatory requirements. Each component is traced to specific requirements in both civilian and defence domains, supporting dual-use compliance planning. The results presented in Section 5 tables ( Tables 6– 17) are illustrative and do not represent an exhaustive set of requirements.

**Table 6.** Espionage detection system Component Mapping to Regulatory Requirements.

System Component	Data/Activity	Applicable Civilian Requirements	Applicable Defence Requirements
USB Camera	Captures images of room occupants (potential biometric data)	CIV-DESIGN-R1: DPIA required; data minimisation in image capture design	DEF-DESIGN-R1: DPIA required; privacy safeguards embedded in design
AI Detection Module	Processes visual data to identify unauthorised photography attempts	CIV-DESIGN-R2: Secure architecture; hardened firmware; secure boot	DEF-DESIGN-R2: NATO security controls; multi-layered access controls
Screen Interface	Ethernet connection; displays classified content	CIV-DESIGN-R2: Encryption architecture for data in transit	DEF-DESIGN-R2: NSP physical security requirements; TEMPEST considerations
5G Transmission	Transmits personal data (images, location) to remote server	CIV-USAGE-R4: Encrypted transmission; audit logging	DEF-USAGE-R4: Secure encrypted channels (Art. 5 [8])
Remote Monitoring Station	Receives alerts; stores images; enables personnel access	CIV-USAGE-R3: Breach notification within 72 hours; data subject rights	
		CIV-USAGE-R4: Access controls; audit logging; vulnerability management	DEF-USAGE-R4-ART3: Detection and reporting mechanisms
Dedicated Personnel	View captured images; make response decisions	CIV-USAGE-R3: Transparency notices; purpose limitation	DEF-USAGE-R4-ART7: Threat intelligence sharing across NATO entities

### 5.3. Analysis of cross-domain compliance patterns

The component mapping reveals two distinct patterns in how regulatory requirements apply across civilian and defence deployment contexts.

***Pattern 1: Divergent Requirements (Domain-Specific Security Frameworks)***

Security-related requirements (R2 and R4) diverge significantly between domains. Civilian deployments rely on EU cybersecurity frameworks, while defence deployments must satisfy NATO-specific standards (see Table 7).

**Table 7.** Divergent requirements regarding design and usage security.

Requirement	Civilian Framework	Defence Framework	Key Differences
R2 (Design, Security)	GDPR Article 32, NIS2 Article 21	NATO NIAP, NATO NSP	Civilian: commercial encryption standards (TLS, AES). Defence: NATO-approved cryptography; personnel security clearances; physical security requirements
R4 (Usage, Security)	NIS2 Article 21, GDPR Article 32	NATO Cyber Defence Policy	Civilian: report to national supervisory authorities. Defence: secure encrypted channels; threat intelligence sharing across NATO entities

*Key finding:* A dual-use system cannot simply apply civilian security standards to defence deployments. The ontological framework captures this divergence by linking R2 and R4 to different regulatory sources depending on the domain.

***Pattern 2: Additive Requirements (Defence Builds Upon Civilian)***

In systems that are developed for use in both civilian and defence domains, defence deployments do not replace civilian requirements – they add to them. An Espionage detection system deployed in a NATO facility must satisfy:

• National law (R1)
• All applicable NIS2 requirements (civilian baseline) plus
• NATO-specific requirements (NIAP, NSP, Cyber Defence Policy)
• Potential TEMPEST certification for electromagnetic emission security

Table 8 effectively delineates these distinctions.

**Table 8.** Requirements mapped to compliance layer.

Compliance Layer	Requirements
EU Data Protection	CIV-R1, CIV-R3 (GDPR)
EU Cybersecurity	CIV-R2, CIV-R4 (NIS2, GDPR Art. 32)
NATO Information Assurance	DEF-R2 (NIAP, NSP)
NATO Operational Security	DEF-R4, DEF-R4-ART3, DEF-R4-ART5, DEF-R4-ART7
Certification Standards	TEMPEST, STANAG (where applicable)

*Key finding:* The regulatory burden for defence deployment is cumulative, not substitutive. The ontological framework supports this through explicit domain tagging ( *appliesToDomain*), enabling queries that retrieve all applicable requirements for a given deployment context.

### 5.4. Validation of framework capabilities

The case study validates four key capabilities of the ontological framework, supported by recent advancements in semantic modelling and automated compliance.

***Capability 1: Dual-Use Coverage***

The framework demonstrates feasibility of Dual-Use Coverage by successfully capturing 11 requirements across seven regulatory sources (see Table 9). This dual-domain approach is increasingly critical as AI-driven maritime and critical infrastructure systems now operate in “omni-use” environments where offensive and defensive capabilities overlap ( Cichocki, 2025).

**Table 9.** Regulatory requirements across civilian and defence domains.

Metric	Result
Civilian requirements populated	4 (CIV-R1 through CIV-R4)
Defence requirements populated	4 (DEF-R1 through DEF-R4)
Defence sub-requirements	3 (DEF-R4-ART3, DEF-R4-ART5, DEF-R4-ART7)
Total requirements captured	11
Regulatory sources represented	7 (GDPR, NIS2, NIAP, NSP, NATO Cyber Defence Policy, national laws, TEMPEST)

*Validation evidence*: SPARQL Query A (Section 3.1, Step 6) successfully retrieved all 8 primary requirements across both domains in a single query, confirming structural completeness.

***Capability 2: Lifecycle Phase Organization***

The framework allocates privacy-by-design to the design phase (see Table 10). This is enabled by ontology-driven context-aware frameworks that dynamically adjust privacy decisions and obfuscation levels based on entity sensitivity, such as masking bystander faces in real time while maintaining the security intelligence of the monitor’s surroundings ( Topalli & Badii, 2025).

**Table 10.** Systems development life cycle phases supported by design principles.

Lifecycle Phase	Privacy-Related	Security-Related	Design Principle
Design and Development (pre-deployment)	R1 (both domains)	R2 (both domains)	Privacy-by-design (GDPR Art. 25, national law)
Usage and Maintenance (post-deployment)	R3 (civilian domain)	R4 (both domains)	Security-by-design (GDPR Art. 32)

*Validation evidence*: The espionage detection system component mapping ( Table 10) demonstrates that design-phase requirements (R1, R2) apply to system architecture decisions (camera design, AI module, encryption architecture), while usage-phase requirements (R3, R4) apply to operational activities (transmission, monitoring, personnel access).

The use of four primary requirement nodes per domain (R1–R4) in this study reflects a minimal structural template designed to demonstrate methodological feasibility rather than a limitation of the ontological framework. The symmetrical organisation across domains (design/privacy, design/security, usage/privacy, usage/security) provides a controlled baseline that enables systematic cross-domain comparison and validation through SPARQL queries. In practice, the framework is not constrained to four requirements per lifecycle phase or domain. Additional requirement instances can be introduced at any level of granularity, including article-level, paragraph-level, or control-level decomposition, without altering the structural integrity of the ontology. The current configuration serves as a proof-of-concept instantiation that illustrates how regulatory obligations can be categorised, decomposed, and aligned across civilian and defence contexts. The scalability of the model derives from its class-property architecture rather than from the number of instantiated requirement nodes.

***Capability 3: Cross-Domain Comparison***

The framework enables systematic alignment of civilian and defence requirements (see Table 11). Research in 2025 demonstrates that while security frameworks diverge, hybrid ANN-ISM modelling can improve the accuracy of 5G network security systems by identifying interdependencies between disparate regulatory process areas ( Khan et al., 2025).

**Table 11.** Civilian and defence requirements compared.

Position	Cross-Domain Query Result	Finding
R1 (Design, Privacy)	Civilian: GDPR Articles 25, 35; Defence: DPIA and Privacy by Design	DPIA is required, but legal basis differs
R2 (Design, Security)	Civilian: GDPR/NIS2; Defence: NIAP/NSP	Divergent frameworks – different security standards apply
R3 (Usage, Privacy)	Civilian: GDPR Article 33	Applicable only in civilian domain
R4 (Usage, Security)	Civilian: NIS2; Defence: NATO CDP	Divergent frameworks – different reporting and operational security

*Validation evidence:* SPARQL Query B (Section 3.1, Step 6) successfully paired civilian and defence requirements by structural position, enabling the comparative analysis shown in Table 11.

***Capability 4: Hierarchical Decomposition***

High-level requirements, such as NATO Cyber Defence Compliance, are decomposed into article-specific sub-requirements (see Table 12). This decomposition facilitates the use of SHACL constraints and logical reasoners (e.g., HermiT) to automate the validation of system specifications against complex regulatory mandates, such as the High-Risk Supplier management required by the Connecting Europe Facility ( Paskauskas, 2025).

**Table 12.** Decomposition of high-level requirements: traceability.

Parent Requirement	Sub-Requirements	Regulatory Source
DEF-USAGE-R4 (NATO Cyber Defence Compliance)	DEF-R4-ART3: Detection mechanisms	Article 3, Paragraph 2
	DEF-R4-ART5: Secure incident reporting	Article 5, Paragraph 8
	DEF-R4-ART7: Threat intelligence sharing	Article 7, Paragraph 6

*Validation evidence*: The sub-requirements map directly to espionage detection system functions:

• DEF-R4-ART3 → AI detection module and camera system
• DEF-R4-ART5 → 5G encrypted transmission to remote monitoring station
• DEF-R4-ART7 → Integration with NATO threat intelligence networks (for defence deployments)

Summary of validation results is presented in Table 13.

**Table 13.** Validation results.

Capability	Validated	Evidence
Dual-use coverage	✓	11 requirements across 7 regulatory sources
Lifecycle phase organization	✓	Requirements correctly mapped to design vs. usage phases
Cross-domain comparison	✓	SPARQL queries successfully pair and compare requirements
Hierarchical decomposition	✓	NATO CDP decomposed to article-level sub-requirements

The case study confirms that the ontological framework can represent the regulatory compliance requirements for a dual-use AI system, support systematic comparison across deployment contexts, and enable traceability from legal obligations to system components.

### 5.5. Practical implications for system development

The validated framework yields concrete guidance for developers navigating the “additive” burden of dual-use compliance.

***Implication 1: Single Design, Dual Compliance Paths***

A system can be developed as a single technical platform but must support subset configurations. In 5G contexts, this requires semantic interoperability layers that allow the system to interpret information exchange consistently across heterogeneous cloud and edge environments ( Brabra et al., 2016). Thus, the espionage detection system can be developed as a single technical platform, but it must satisfy different compliance paths depending on the deployment context (see Table 14).

**Table 14.** Espionage detection system as a single technical platform.

Development Decision	Civilian Deployment	Defence Deployment
Encryption standard	Commercial TLS/AES (GDPR Art. 32, NIS2 Art. 21)	NATO-approved cryptographic modules
Access control	Role-based access with audit logging	Security clearance verification; need-to-know enforcement
Incident reporting	National supervisory authority (within 72 hours)	Secure encrypted channels to NATO entities
Data retention	Defined by DPIA; subject to erasure requests	May be extended for security evidence; erasure rights potentially limited
Certification	CE marking; NIS2 compliance attestation	TEMPEST certification; STANAG compliance

*Recommendation*: Design the system architecture to accommodate the more stringent defence requirements, enabling civilian deployment as a subset configuration.

***Implication 2: Unified Knowledgebase***

Developers can use a single RDF-based knowledge base to generate deployment-specific documentation (see Table 15). This reduces the “interpretative burden” on smaller entities, who often face disproportionate GDPR enforcement risks despite the lack of direct correlation between violation severity and fine amounts ( Orlando & Santoro, 2025).

**Table 15.** Compliance documentation as regards Civilian-Defence deployment.

Documentation	Civilian Deployment	Defence Deployment
Regulatory basis	GDPR, NIS2 Directive	NIAP + NSP + NATO CDP
Applicable requirements	CIV-R1, CIV-R2, CIV-R3, CIV-R4	DEF-R1, DEF-R2, DEF-R4 (+ sub-requirements)
Certification evidence	NIS2 compliance; GDPR accountability documentation	TEMPEST certificate; NATO accreditation
Audit trail	Access logs; breach register; DPIA	Above + threat intelligence sharing records

*Recommendation*: Use SPARQL queries to generate deployment-specific compliance checklists. The query FILTER(?domain = rc: CivilianDomain) or FILTER(?domain = rc: DefenceDomain) retrieves only the applicable requirements for each context.

***Implication 3: Procurement and Commercialisation Strategy***

For the espionage detection system developer, the framework informs market positioning, as shown in Table 16.

**Table 16.** Market positioning.

Market Segment	Compliance Requirements	Supervisory Pathway
Corporate sector	GDPR, NIS2 (if critical infrastructure)	National authority registry control
Government (civilian)	GDPR, NIS2, national cybersecurity law	National authority accreditation
Financial institutions	GDPR, DORA	Regulatory compliance supervision
NATO/Defence	GDPR + full NATO framework	TEMPEST certification; NATO accreditation

*Recommendation*: Develop compliance packages for each market segment, using the ontological framework to generate segment-specific requirements documentation. The civilian package represents the baseline; defence package adds NATO-specific requirements.

### 5.6. Key case study findings

The espionage detection system case study validates the proposed ontology-based methodology for regulatory compliance requirements specification in dual-use AI systems. The key findings are summarised below:

1) *The methodology produces an integrated and queryable compliance framework.* The six-step methodology yielded an RDF/Turtle ontology containing 11 regulatory requirements across civilian and defence domains, derived from 7 regulatory sources. The framework was successfully deployed in Stardog Cloud and validated through SPARQL queries that retrieved, compared, and analyzed requirements across domains.
2) *Structural parallelism enables systematic cross-domain analysis.* The consistent organization of requirements by lifecycle phase (design/development vs. usage/maintenance) and category (privacy-related vs. security-related) in both domains enables direct comparison. SPARQL queries can automatically pair civilian and defence requirements by structural position (e.g., CIV-DESIGN-R1 ↔ DEF-DESIGN-R1), revealing where requirements align and where they diverge.
3) *Security frameworks diverge by domain.* Security-related requirements (R2, R4) are sourced from different regulatory frameworks depending on deployment context. Civilian deployments rely on GDPR Article 32 and NIS2 Directive; defence deployments require compliance with NATO Information Assurance Policy, NATO Security Policy, and NATO Cyber Defence Policy. The framework captures this divergence through domain-specific derivedFrom relationships.
4) *Defence compliance is additive, not substitutive.* For the systems that are deployed in both civilian and defence domains, defence deployments must satisfy all applicable civilian requirements plus NATO-specific obligations. The ontological framework supports this cumulative model through explicit domain tagging (appliesToDomain), enabling queries that retrieve the set of requirements for a given deployment context.
5) *The framework supports practical compliance planning.* The validated ontology yields actionable guidance for system developers, enabling them to trace system architecture to legal obligations, identifying required certification, etc. More details are provided in Table 17.

**Table 17.** Benefits of a validated ontological framework.

Application	Benefit
Component-to-requirement mapping	Traceability from system architecture to legal obligations
Deployment-specific queries	Generate civilian or defence compliance checklists from single knowledge base
Conflict identification	Reveals tensions between GDPR transparency and defence classification
Certification pathway planning	Identifies required certifications for each market segment
DPIA scoping	Universal entry point for both civilian and defence deployments even if derived from different legal sources

### 5.7. Case study summary

The espionage detection system case study demonstrates that the proposed ontology-based methodology can accomplish the following:

• Represent regulatory requirements from multiple, overlapping legal frameworks.
• Organize requirements by lifecycle phase and category to support privacy-by-design and security-by-design.
• Enable systematic comparison across civilian and defence deployment contexts.
• Support hierarchical decomposition for precise traceability to regulatory articles.
• Generate practical compliance guidance for dual-use technology developers.

These capabilities address the limitations identified in Section 2.5: the framework explicitly considers multiple regulations and provides a structured approach for dual-use technology compliance that maintains traceability between legal requirements and technical specifications.

## 6. Summary and future work

This section provides the summary of the paper, highlighting major contributions and limitations, and directions for future work.

### 6.1. Summary

This paper addressed the challenge of specifying regulatory compliance requirements for AI systems operating in dual-use contexts, where civilian and defence deployment scenarios impose overlapping, divergent, and sometimes conflicting obligations. As the EU digital regulatory ecosystem expands – encompassing the AI Act, GDPR, NIS2 Directive, and sector-specific frameworks – and as dual-use technologies increasingly operate at the intersection of civilian and defence domains, systematic approaches to compliance specification become essential.

We proposed a six-step ontology-based methodology that enables: Structured scoping of applicable regulations across civilian and defence domains (step 1); Conceptual modelling using lifecycle phases and requirement categories (step 2); Formal ontology design and implementation in RDF/Turtle (step 3); Systematic population of domain-specific requirements (step 4); Deployment in knowledge graph platforms for visualisation and exploration (step 5); SPARQL-based validation and cross-domain analysis (step 6).

The methodology was validated through a case study applying the framework to the espionage detection system – an AI-powered dual-use technology designed to detect unauthorised photographing of classified information.

### 6.2. Contributions

This paper makes several contributions to the emerging literature on AI governance, regulatory technology (RegTech), and ontology-based requirements engineering, described in Table 18. It proposes a regulatory compliance framework for dual-use technologies, which integrates privacy-by-design and security-by-design principles. Proposed framework has been validated using a case study. Furthermore, practical guidance for developers has been provided.

**Table 18.** A summary of contributions.

Contribution	Description
Dual-use compliance framework	A structured ontological approach that explicitly models regulatory requirements for technologies deployable in both civilian and defence contexts, validated across maritime, energy, and telecommunications sectors ( Cichocki, 2025; Ndiaye et al., 2026)
Lifecycle-phase organization	Integration of privacy-by-design (GDPR Article 25) and security-by-design (GDPR Article 32) principles, technically supported by ontology-driven context-aware frameworks for real-time video obfuscation ( Topalli & Badii, 2025)
Cross-domain analysis capability	SPARQL queries that systematically compare civilian and defence requirements, enhanced by hybrid ANN-ISM modelling to identify security interdependencies in 5G networks ( Khan et al., 2025)
Validated methodology	A replicable six-step process, including SHACL constraints and logical reasoners (e.g., HermiT) to automate the detection of compliance gaps in high-risk infrastructure ( Paskauskas, 2025; Ndiaye et al., 2026)
Practical compliance guidance	Actionable findings for developers, including Re-Identifiability Indices (RII) for vision systems and conflict resolution strategies for “omni-use” deployment tensions ( Topalli & Badii, 2025; Cichocki, 2025)
Emerging Tech Roadmap	Extensions for 6G security (ES3A), decentralized Blockchain compliance (BAML), and federated learning, addressing the shift toward autonomous, agentic AI governance ( Duan et al., 2025; Khan et al., 2025)

### 6.3. Scope of automation

The proposed framework automates the structuring, alignment, and retrieval of regulatory compliance requirements rather than the legal interpretation process itself. Regulatory texts are initially translated into ontological representations through expert-driven abstraction. Once formalised, however, compliance applicability, cross-domain comparison, lifecycle allocation, and requirement decomposition become computationally executable. The automation therefore operates at the level of compliance configuration and validation, not at the level of autonomous legal reasoning.

### 6.4. Limitations

The current work has several limitations that should be acknowledged:

• *Scope of regulatory coverage.* The ontology captures a subset of applicable regulations (GDPR, NIS2, NATO policies) and only a very limited number of selected requirements for illustrative purposes. Additional frameworks – including the EU AI Act risk classification, Cyber Resilience Act, sector-specific regulations (e.g., DORA for financial services), and national transposition laws – were identified but not fully modelled. Additionally, the broader framework of international law, including international humanitarian and human rights law, was referenced but not fully analysed to be operationally included in the proposed framework.
• *Granularity of requirements.* Requirements were specified at a summary level (R1–R4 per domain) with selective decomposition (DEF-R4 sub-requirements). A production compliance system would require more granular specification at the article and paragraph level across all applicable regulations.
• *Single case study.* Validation was conducted using one dual-use technology (espionage detection system) and only on a very high level without going into the details of factual deployment in defence scenarios. Generalizability to other dual-use AI systems – such as autonomous vehicles, medical AI, or critical infrastructure monitoring – requires additional case studies.
• *Static representation.* The current ontology captures requirements at a point in time. Regulatory frameworks evolve; the AI Act implementing standards are still under development by CEN-CENELEC JTC 21. Mechanisms for ontology versioning and update propagation were not addressed.
• *Expert validation.* While the framework was validated through SPARQL queries demonstrating structural completeness and cross-domain comparison capability, formal validation with legal experts, compliance officers, and certification bodies was not conducted within the scope of this study.

### 6.5. Future work

Building on the contributions and addressing current limitations, the following directions for future research are identified:

• *Extended Regulatory Coverage and Granularity*: Expand the ontology to include article-level mappings for the EU CRA and CEN-CENELEC JTC 21 standards. This includes integrating co-assurance frameworks that harmonize safety standards (e.g., IEC 61508) with EU-wide legislative requirements ( Ndiaye et al., 2026).
• *Automated Compliance with SHACL and Reasoners*: Further develop SHACL (Shapes Constraint Language) rules to validate system specifications. Future iterations will leverage logical reasoners to automate the detection of compliance gaps, particularly for high-risk infrastructure components like SDN and cloud-based 5G subsystems ( Paskauskas, 2025).
• *Integration of Decentralized and Privacy-Preserving Technologies*: Investigate the use of Blockchain Hyperledger and Federated Learning (FL) to enable secure, privacy-preserving financial and security compliance ( Khan et al., 2025). This addresses the challenge of data silos while ensuring a consensus-driven feedback loop for security models.
• *Evolution Toward 6G and Edge Intelligence*: Adapt the framework for 6G networks by incorporating Smart Service-based Security Architectures (ES3A) ( Duan et al., 2025). Research will focus on edge–cloud IoT frameworks that perform real-time, privacy-preserving analytics ( Xia, 2025).
• *Semantic Interoperability in Multi-Cloud Environments*: Enhance the ontology to support federated-fog computing. This includes developing semantic layers that mitigate latency and optimize computational resources for real-time IoT applications across diverse organizations ( Huaranga-Junco et al., 2024).
• *Generative AI and Large Language Model Support*: Explore the use of LLMs and Retrieval-Augmented Generation (RAG) to assist in extracting requirements from emerging 2025/2026 regulations. Future work will evaluate if these systems can overcome the lack of true semantic understanding in automated governance contexts ( Wang, 2025).

### 6.6. Concluding remarks

The growing complexity of the EU digital regulatory ecosystem, combined with the increasing prevalence of dual-use AI technologies, demands systematic approaches to compliance specification. Manual interpretation of overlapping regulations is error-prone, resource-intensive, and difficult to maintain as frameworks evolve.

Ontology-based methods offer a pathway to a structured, machine-readable compliance specification that supports traceability, cross-domain analysis, and automated validation. By representing regulatory requirements in a formal knowledge graph, organizations can navigate the intersection of civilian and defence obligations more systematically – reducing compliance risk while enabling innovation in dual-use technology development.

The methodology and framework presented in this paper represent an initial step toward automated regulatory compliance for AI systems. As the EU AI Act implementation progresses and technical standards mature, ontological approaches will become increasingly valuable for operationalising abstract legal norms within complex AI systems.

## Software availability

Source code available from: https://github.com/SecOntologyLab/regulatory-compliance-ontology

License: MIT.

Archived software available from: https://doi.org/10.5281/zenodo.18763491 ( Paskauskas, 2026)

The knowledge graph was hosted and SPARQL queries were executed using Stardog Cloud ( https://www.stardog.com/), latest stable release accessed February 2026.

Interactive visualisations were developed using D3.js ( https://d3js.org/).

## Ethics and consent

Ethical approval and consent were not required.

## Data Availability

The data that supports the findings of this study are openly available in repository: https://doi.org/10.5281/zenodo.18763491 ( Paskauskas, 2026) Data is available under the terms of the Creative Commons Attribution 4.0 International. Repository contains ontology files (TTL), SPARQL query library, methodology documentation, and interactive visualisations required to reproduce the reasoning patterns described in the manuscript. Files are provided in open, non-proprietary formats (Turtle/TTL, SPARQL, HTML) conforming to W3C Semantic Web standards. All artefacts required to reproduce the ontological reasoning patterns described in the manuscript are included in the repository. No additional extended data beyond the repository contents.
