# Supplementary material for: Automated regulatory compliance for AI systems in the security domain: The case of dual-use deployment
Source: Open Res Eur. 2026 Mar 28;6:83. [Version 1] doi: 10.12688/openreseurope.23137.1 (PMC13231260; doi:10.12688/openreseurope.23137.1)
Supplement: Peer review history

# Reviewer report: Reviewer response for version 1

**DOI:** 10.21956/openreseurope.25029.r73927

**Published:** 2026-05-27

**Authors:** Aryendra Dalal

**Affiliations:** 1 Middle Georgia State University, Macon, Georgia, USA

This paper proposes a six-step ontology-based methodology for specifying regulatory compliance requirements for dual-use AI systems, implemented as an RDF/Turtle knowledge graph and validated through a case study involving an AI-powered espionage detection system. The regulatory scope spans EU instruments (AI Act, GDPR, NIS2) and NATO frameworks, with SPARQL queries enabling cross-domain compliance comparison.

**Is the work clearly and accurately presented and does it cite the current literature? — Partly** The paper is well-written overall and references recent literature through 2026. However, the academic positioning needs tightening. The related work section lists prior ontological frameworks as bullet points without meaningfully engaging with how they fall short or how this work specifically improves upon them. That gap weakens the novelty claim considerably.

**Is the study design appropriate and does the work have academic merit? — Yes** Using ontology-based requirements engineering for dual-use compliance is a reasonable and timely choice. The layered civilian-plus-defence compliance model captures something genuinely important that simpler approaches miss. The six-step structure is coherent, and the espionage detection system is a credible test case for the methodology.

**Are sufficient details of methods and analysis provided to allow replication by others? — Partly** The SPARQL queries are included and the GitHub/Zenodo links are provided, which is commendable. That said, the ontology class hierarchy is not visually presented in a way that would allow an independent team to reconstruct it without difficulty. The process by which regulatory text is translated into ontological representations is described only at a high level — this expert-driven abstraction step is arguably the most consequential part of the methodology and deserves considerably more transparency. A worked example mapping a specific regulatory article to an ontological class would help significantly.

**Statistical Analysis — Not Applicable**

**Are all source data underlying the results available? — Partly** The repository exists and is cited, but the full query set used to generate the tables in Section 4 is not completely documented within the manuscript itself. Readers should not have to navigate externally to verify core results.

**Are the conclusions drawn adequately supported by the results? — Partly** The four validated capabilities (dual-use coverage, lifecycle organization, cross-domain comparison, hierarchical decomposition) are all reported as successfully confirmed, which reads somewhat circular — the framework is introduced, populated with requirements the authors themselves defined, then queried to confirm those same requirements are present. This is structural completeness testing, not genuine validation against an independent benchmark or real deployment scenario. Formal engagement with legal or compliance experts would substantially strengthen the validation claim. The paper also acknowledges static representation as a limitation of existing approaches but then lists it again as a limitation of its own system, without addressing that tension.

**Minor Points** Two typographical errors need correcting: "focuses number of" on page 9 and "as included in. the table" on page 21. Several sections would read better as continuous prose rather than bulleted lists, particularly the future work section.

Is the study design appropriate and does the work have academic merit?

Yes

Is the work clearly and accurately presented and does it cite the current literature?

Partly

If applicable, is the statistical analysis and its interpretation appropriate?

Not applicable

Are all the source data underlying the results available to ensure full reproducibility?

Partly

Are the conclusions drawn adequately supported by the results?

Partly

Are sufficient details of methods and analysis provided to allow replication by others?

Partly

Reviewer Expertise:

Cybersecurity

I confirm that I have read this submission and believe that I have an appropriate level of expertise to confirm that it is of an acceptable scientific standard, however I have significant reservations, as outlined above.

# Reviewer report: Reviewer response for version 1

**DOI:** 10.21956/openreseurope.25029.r72142

**Published:** 2026-05-25

**Authors:** Florentina-Loredana Dragomir-Constantin

**Affiliations:** 1 National Defense University Carol I, Bucharest, Romania

The article “Automated regulatory compliance for AI systems in the security domain: The case of dual-use deployment” addresses an important and timely research topic situated at the intersection of artificial intelligence, information systems, national security, and dual-use technologies. The paper proposes an ontology-based methodology for specifying requirements applicable to AI systems that may be developed or deployed across both civilian and defence-related contexts. The material evaluated presents a coherent conceptual and methodological framework, supported by a case study focused on an AI-powered espionage detection system. The main contribution of the article consists in its attempt to formalize complex legal, technical, and security-related requirements into machine-readable representations through knowledge graphs, RDF/Turtle modelling, SPARQL-based analysis, and semantic validation.

 The paper is relevant both academically and practically, as it addresses the growing need for structured and traceable approaches to AI systems operating in sensitive and security-relevant environments.

 Q1: Is the work clearly and accurately presented and does it cite the current literature?

 Response: Yes.

 The work is clearly and accurately presented. The article is logically structured and introduces the reader to the complexity of AI systems that may operate across civilian and defence contexts. The discussion of overlapping obligations, system lifecycle phases, and dual-use deployment scenarios is generally well organized and easy to follow. The paper cites current and relevant literature, particularly in relation to the EU AI Act, digital regulation, AI governance, dual-use technologies, and ontology-based approaches. The references support the overall argument and demonstrate that the authors are engaging with a contemporary and evolving research area. For future development, the paper could further strengthen its academic framing by positioning the proposed methodology more explicitly in relation to existing debates on dual-use AI, national security applications, and ontology-based requirements engineering.

 Q2: Is the study design appropriate and does the work have academic merit?

 Response: Yes.

 The study design is appropriate for the objectives of the article. The ontology-based methodology is suitable for organizing complex and overlapping requirements and for making them more transparent, traceable, and reusable. The six-step methodological structure provides a clear pathway from regulatory scoping to ontology development, knowledge graph deployment, and SPARQL-based validation. The work has academic merit because it addresses a relevant interdisciplinary problem that connects artificial intelligence, information systems, national security, security governance, and dual-use technologies. The article contributes to an emerging area of research by proposing a structured approach for translating complex requirements into system-level specifications. The case study also adds value by illustrating how the proposed framework may be applied to a concrete AI system operating in a sensitive security-related environment.

 Q3: Are sufficient details of methods and analysis provided to allow replication by others?

 Response: Partly.

 The article provides a clear overview of the proposed methodology and presents the main technical elements used in the framework, including RDF/Turtle, knowledge graphs, SPARQL queries, and semantic validation. These elements provide a useful basis for understanding how the approach may be implemented. However, full replication would benefit from additional methodological detail. In particular, the authors could further explain how legal, policy, and security-related texts are interpreted and transformed into ontology classes, properties, relations, and constraints. More examples of mappings between requirements and ontology elements would also improve transparency. For future versions of the work, it would be useful to include a more detailed explanation of the role of experts in the validation process, the criteria used for assessing the ontology, and the steps required for another researcher to reproduce the knowledge graph and validation results.

 Q4: If applicable, is the statistical analysis and its interpretation appropriate?

 Response: Not applicable.

 The article does not rely on statistical analysis. It is primarily a conceptual, methodological, and technical contribution based on ontology modelling and case-study validation. Therefore, this criterion is not applicable.

 Q5: Are all the source data underlying the results available to ensure full reproducibility?

 Response: Yes.

 The article provides sufficient indications regarding the availability of the underlying resources, including ontology-related files and SPARQL queries. This supports transparency and contributes positively to the reproducibility of the study. For future improvement, the authors may consider offering more detailed documentation of the ontology files, query library, validation process, and knowledge graph structure. This would make the proposed framework more accessible to researchers and practitioners interested in applying or extending the methodology.

 Q6: Are the conclusions drawn adequately supported by the results?

 Response: Yes.

 The conclusions are adequately supported by the methodology and the case study presented in the article. The authors show that an ontology-based approach can support the structuring, mapping, and analysis of requirements for AI systems deployed in dual-use and security-related contexts. The conclusions are consistent with the results of the study and reflect the potential value of the proposed framework. For future development, the article could further strengthen its conclusions by discussing in more detail the practical limits of the approach, including scalability, ontology maintenance, updates following legal or policy changes, and possible conflicts between civilian and defence-related requirements. The article is timely, relevant, and well aligned with current challenges in artificial intelligence, information systems, national security, and dual-use technology governance. The proposed ontology-based methodology is a valuable contribution and has clear potential for further academic and practical development.

 For future improvement, the authors may consider the following points:

 The academic contribution could be clarified further by explaining more explicitly how the proposed framework differs from and improves upon existing ontology-based or requirements engineering approaches.

 The discussion of dual-use AI could be expanded by engaging more deeply with the academic literature on national security, defence applications, and the relationship between civilian and military uses of AI systems.

 The methodology could be strengthened by providing more detail on how textual requirements are interpreted, selected, categorized, and translated into ontology components.

 The validation section could be improved by specifying clearer criteria, thresholds, or indicators for evaluating the functionality and reliability of the proposed framework.

 The article could include a more detailed discussion of scalability, especially regarding the future updating of the ontology when regulations, standards, or security policies change.

 The presentation could be refined by replacing some bullet-point sections with continuous analytical discussion and by correcting minor wording issues.

 These recommendations are offered as constructive suggestions for future refinement. They do not diminish the overall quality, originality, or relevance of the work.

 Final Recommendation

 Approved.

              The manuscript represents an important and timely contribution to the study of AI systems in the security domain, with particular relevance for information systems, national security, and dual-use technologies. The material evaluated demonstrates methodological coherence, academic relevance, and practical potential. Although some aspects could be developed further in future versions, particularly the methodological details, validation process, and academic positioning, these are recommendations for improvement rather than reasons to withhold approval. Overall, the article is well conceived, relevant, and suitable for approval.

Is the study design appropriate and does the work have academic merit?

Yes

Is the work clearly and accurately presented and does it cite the current literature?

Yes

If applicable, is the statistical analysis and its interpretation appropriate?

Not applicable

Are all the source data underlying the results available to ensure full reproducibility?

Yes

Are the conclusions drawn adequately supported by the results?

Yes

Are sufficient details of methods and analysis provided to allow replication by others?

Partly

Reviewer Expertise:

Information systems, artificial intelligence, national security, dual-use technologies, machine learning, decision theory.

I confirm that I have read this submission and believe that I have an appropriate level of expertise to confirm that it is of an acceptable scientific standard.

# Response

**Published:** 2026-05-26

**Authors:** Giedre Sabaliauskaite

**Affiliations:** Mykolas Romeris University, Vilnius, Vilnius County, Lithuania

We thank the reviewer for the careful assessment of our manuscript and for the constructive recommendations provided. We appreciate the approval of the article and acknowledge the suggestions regarding the academic positioning, dual-use AI framing, methodological detail, validation process, scalability, ontology maintenance, and presentation. We will take these points into account in preparing the revised version of the manuscript, particularly by clarifying the methodology, strengthening the discussion of validation and limitations, and improving the presentation where appropriate.

# Reviewer report: Reviewer response for version 1

**DOI:** 10.21956/openreseurope.25029.r72145

**Published:** 2026-05-14

**Authors:** Stefka Schmid

**Affiliations:** 1 Aalto University, Helsinki, Finland

The work titled “Automated regulatory compliance for AI systems in the security domain: The case of dual-use deployment” contributes to an important and timely empirical topic by presenting an ontology for automated compliance of dual-use AI systems. At the same time, the work should connect more to relevant academic debates and situate their case better in the real-life environment of dual-use AI automated compliance. While the development of the ontology is overall understandable, it should be ensured that the approach is systematic and also entails a reflection on the boundaries of the proposed artifact.

**Introduction and Framing**

 The introduction clearly lays out the empirical relevance of the author’s work. In this regard, it would be useful to open with a concrete example of a dual-use system that may need to comply not only with the AI Act but also with defence standards such as NIAP and NSP, as this would immediately ground the paper's subject matter. The academic contribution also needs to be clarified early on, as it is currently not clear what other approaches look like or how this work positions itself against them. While the research gap is laid out, the EU case needs to be better plausibilized; the reader should understand from the outset why it is necessary to focus specifically on this issue. Finally, the conclusion should circle back to this framing with a short note on the critical nature of automated regulatory compliance, referencing the state of the art and explaining why it is useful and promising to focus on this issue despite challenges such as those identified in the paper.

**Academic Contribution: Situating in Scholarship**

 While the empirical relevance of the work is clear, the paper needs to more explicitly address questions of feasibility, potential pitfalls, and the current state of the scholarly debate. The academic contribution requires stronger clarification, particularly regarding how this work differs from and builds upon existing approaches. A significant gap currently lies in the treatment of ontological frameworks for automating regulatory compliance: these are presently only listed as bullet points, with no substantive engagement with how the authors assess this body of work or how it connects to automated compliance tools for both civilian and military contexts. This is central to the paper’s academic relevance and must be situated more carefully within the relevant scholarships. Related to this, the paper should address how well existing frameworks have performed, what their level of technological readiness is, and how differences between civilian, military, and dual-use automated compliance systems are reflected in the literature. The current limitations of other approaches should be explained explicitly, with a clear account of how this work specifically addresses them. The claim that existing AI governance compliance approaches rely on static documentation needs to be supported with references, and the added value of automated validation should be illustrated accordingly. Further, static representation is criticized as a limitation of existing approaches, yet it is subsequently named as a limitation of the presented work.

**Dual-Use Concept and AI Classification**

 The general definition of dual use is acceptable, but should be accompanied by references to the scholarly debate on this concept. A strength of the paper is its emphasis on AI being inherently dual-use. This is an important intervention, given that much existing scholarship foregrounds the “general purpose” character of AI in a way that treats it as neutral and thereby overlooks the different regulatory regimes that may apply to security-critical domains. However, when the paper criticizes the “strict binary logic of dual use” as something that slows down innovation, this requires clarification: given that the paper has already argued that AI is inherently dual-use, the critique here seems to be directed more at the institutional separation of civilian and military spheres than at the fact that systems can serve both purposes. Even though the link between the high-risk classification and dual-use systems is understandable, the relationship between dual-use and high-risk AI systems also needs to be elaborated, particularly to justify why high-risk AI is introduced in section 2.3 and how it connects to the dual-use issue. The paper should additionally engage with national legislation that mandates the separation of civilian and military spheres (e.g. in Germany). When the espionage detection system is introduced as an example, its dual-use character should be justified immediately by explaining the range of contexts in which such a system might be deployed. Comparable systems that exist should be referenced, given that the example system apparently does not.

**EU Regulatory Framework**

 The EU regulatory debate and its dynamics should also be situated in relation to national decision-making processes and dynamics. In discussing national data protection, examples beyond the mere national implementation of GDPR should be included. The claim that “in the legal scholarship, it is emerging that real applicability of the exemptions included under certain EU regulations such as the GDPR or AI Act is uncommon in practice, one of the reasons being that governments and militaries often tend to rely on private companies to develop AI” needs to be unpacked and explained more clearly. The list of obligations for high-risk AI systems should be properly referenced, as it appears not to be the authors’ own formulation. The use of CJEU rulings as examples should also be better justified through written explanation rather than bullet points. Finally, the paper raises the important point that it requires “extreme cautiousness to capture all possible scenarios so that no AI system component slips through the regulatory gaps” but does not explain how this is typically achieved, i.e., whether humans are successfully doing this currently, or whether this has become a more pressing concern in light of the EU’s new dual-use strategy and what specifically has changed in that context.

**International Humanitarian Law**

 The claim that the Geneva Conventions of 1949 and their Additional Protocols lay down which actions can be taken during warfare, including when AI is used, requires more careful framing. While this is a reasonable choice, it should be acknowledged that AI is not specifically addressed in the Geneva Conventions; their categories are broad and, where they do not deal with specific weapon systems, they are technology-agnostic. The paper should also engage with the UN Group of Governmental Experts (GGE) and perhaps the REAIM summits. While the ICRC is a very relevant organization, it is a non-governmental organization. More broadly, the paper should consider that not all the regulatory instruments it lists carry equal normative weight. Finally, the factors identified as relevant, such as intended end users, data categories, primary purpose, and geographic location,  should be more clearly connected to what has been established in the preceding discussion of IHL and related frameworks, so that readers understand how these factors were derived and why they matter.

**Defence Standards and Compliance**

 When referencing the NATO Information Assurance Policy and similar instruments in section 2.4, proper citations should be included. The claim that “compliance in defence deployments is, therefore, mainly achieved through accreditation processes and security authorization rather than conformity assessment under EU market legislation” also needs to be explained more clearly, as it is not immediately obvious what this distinction entails in practice and why it matters for the paper’s argument.

**AI Life Cycle**

 With regard to different life cycle phases, the paper could engage with Bode et al.’s recommendations regarding human interaction with decision support systems across different phases. The lifecycle phase organization is a notable strength of the paper, and the issue of dual regulatory coverage across life cycle phases is also important but life cycle phases should be clarified specifically regarding AI in section 2.1. It should also be clarified whether the activities and outputs described in the steps are meant to be followed in chronological order; if so, they should be numbered accordingly.

**Methodology: Ontology Development**

 The paper would benefit considerably from grounding its approach in a recognized methodological framework, for example from information systems research or an established ontology development method. Without this, it is difficult to assess how the work proceeds and to what extent it builds on existing methods. In particular, it is unclear whether steps 1 to 6 are derived from such a method. It should also be clarified whether the first step of defining the technology is intended solely for dual-use contexts, or whether the artifact is meant to be applicable more broadly. The inclusion of domain experts is welcome, but it is currently unclear what kind of participatory approach is being used, how experts are to be included specifically, and the artifact should be introduced as such from the outset. The description of “regulatory texts being initially translated into ontological representations through expert-driven abstraction” is a key methodological step that warrants considerably more explanation. More generally, since the data consists of written legal text that requires interpretive skill and involves variation in formulation, the paper needs to provide concrete examples of how text is analyzed in practice. This includes a comparison with human interpretation to make a convincing case for an automated approach.

**Automated Compliance and Validation**

 The authors acknowledge that “we cannot rely on AI models yet” but then proceed to outline steps for automating compliance without adequately plausibilizing why this endeavor is worthwhile given that caveat. The validation of the artifact needs to be made more transparent: Readers should understand how the validation was conducted and any references to “recent advancements in semantic modelling and automated compliance” that support the validation results should be properly cited. The current validation is problematic in that all capabilities are reported as validated, which risks appearing tautological, as if the system is introduced and then simply confirmed to work. It is essential that validation thresholds, metrics, and criteria are made explicit and intersubjectively verifiable, and that nuances in performance are acknowledged and discussed. The paper should explain not only whether the artifact can be validated but how, drawing on the existing knowledge base to justify the approach. It should also be explained how the automated system ensures that the legal basis, as reflected in the right column of Table 4, is checked adequately. Finally, while expert validation is a valid element, the work could be further strengthened by comparing the proposed artifact against existing approaches. Building on the work, implications are derived. However, currently, it is not clear how the implications presented in the paper are derived, whether from the validation process or from comparison with existing approaches and from which specific finding a specific implication can be formulated.

**Presentation, Minor Points**

 There are two instances of missing words that should be corrected: on page 9, “focuses [on a] number of” and on page 21, “as included in. the table.” The use of CJEU rulings would be better handled through written paragraphs rather than bullet points. Throughout the paper, bullet points should be revised into continuous written text, particularly in the future work section, where it should be made clear which directions of future work follow from which limitations or contributions. The ordering of the final sections should be revised so that findings precede implications, as implications should build on findings. The reference to Lithuania in Table 3 requires explanation, though it could potentially be incorporated into the case study. The identification of applicable defense policy within the case study should also include national legislation. The case study summary in section 5.7 should be removed. Section 6.1 should drop its summary in favor of a focused reflection on the paper’s contribution. The statement that dual-use systems should not implement prohibited functionality may come across as trivial and should either be better justified or reframed. Finally, when maritime and critical infrastructure systems are mentioned, the reader should be clearly informed that these are application fields for dual-use systems.

Is the study design appropriate and does the work have academic merit?

Yes

Is the work clearly and accurately presented and does it cite the current literature?

Partly

If applicable, is the statistical analysis and its interpretation appropriate?

Not applicable

Are all the source data underlying the results available to ensure full reproducibility?

Yes

Are the conclusions drawn adequately supported by the results?

Partly

Are sufficient details of methods and analysis provided to allow replication by others?

Partly

Reviewer Expertise:

dual-use AI, innovation research, technology and international security

I confirm that I have read this submission and believe that I have an appropriate level of expertise to confirm that it is of an acceptable scientific standard, however I have significant reservations, as outlined above.

# Response

**Published:** 2026-05-15

**Authors:** Giedre Sabaliauskaite

**Affiliations:** Mykolas Romeris University, Vilnius, Vilnius County, Lithuania

We thank the reviewer for the detailed assessment of our manuscript and for the constructive comments provided. We acknowledge the concerns raised regarding the framing of the academic contribution, the treatment of dual-use AI, the positioning of the EU regulatory context, the discussion of international humanitarian law and defence standards, and the methodological grounding of the ontology-development and validation process. We will address these points promptly in the revised manuscript by strengthening the introduction and scholarly positioning, clarifying the scope and boundaries of the proposed artefact, expanding the methodological explanation, and revising the discussion of validation, findings, implications, and presentation. We will provide a detailed point-by-point response with the resubmission.

# Reviewer report: Reviewer response for version 1

**DOI:** 10.21956/openreseurope.25029.r73924

**Published:** 2026-05-12

**Authors:** Hewa Majeed Zangana

**Affiliations:** 1 Duhok Technical College, Duhok Polytechnic University, Duhok, Iraq

**1. Article Summary**

 The paper addresses the "compliance gap" created by the overlapping regulatory landscapes for AI systems, specifically focusing on **dual-use deployment**. It explores the intersection of civilian regulations (e.g., EU AI Act, GDPR, NIS2) and defense-specific frameworks (e.g., NATO’s Principles of Responsible Use).

 The authors propose an **Ontology-based Requirements Engineering (ObRE)** approach to automate the identification and mapping of these requirements. By developing a specialized ontology and utilizing semantic reasoning (SPARQL queries), the study demonstrates how to transform abstract legal norms into a machine-readable knowledge graph. The framework is validated through a case study involving an AI-driven espionage detection system, concluding that defense compliance acts as an "additive" layer to civilian requirements rather than a replacement.

**2. Detailed Responses to Review Questions**

**Q1: Is the work clearly and accurately presented and does it cite the current literature?**

- **Response:** **Yes.**
- **Assessment:** The paper is exceptionally well-referenced, citing 2025 and 2026 literature. It effectively contextualizes the EU AI Act within the broader security domain. The narrative flow from the "regulatory thicket" problem to the ontological solution is logical and accessible to both legal and technical audiences.

**Q2: Is the study design appropriate and does the work have academic merit?**

- **Response:** **Yes.**
- **Assessment:** Using ObRE for legal compliance is a scientifically sound choice. The methodology bridges the gap between high-level legal principles and low-level system requirements. The academic merit lies in the specific application to "dual-use" scenarios—a niche that is rapidly becoming critical as defense sectors increasingly adopt commercial AI off-the-shelf (COTS) solutions.

**Q3: Are sufficient details of methods and analysis provided to allow replication by others?**

- **Response:** **Partly.**
- **Critique:** While the six-step methodology is clear, the paper lacks a detailed visualization or schema of the developed ontology. Without seeing the class hierarchy (e.g., how LegalRequirement relates to SystemConstraint), a third party cannot replicate the semantic model accurately.
- **Constructive Feedback:** (See Section 3 for specific actions).

**Q4: If applicable, is the statistical analysis and its interpretation appropriate?**

- **Response:** **Not applicable.**
- **Assessment:** The work is a technical framework and case study; it does not rely on statistical inference.

**Q5: Are all the source data underlying the results available to ensure full reproducibility?**

- **Response:** **Partly.**
- **Critique:** The paper includes snippets of SPARQL queries, but the full RDF/Turtle file (the "source data" for an ontology paper) is not included in the manuscript or a persistent repository link within the text.
- **Constructive Feedback:** (See Section 3 for specific actions).

**Q6: Are the conclusions drawn adequately supported by the results?**

- **Response:** **Yes.**
- **Assessment:** The case study on espionage detection provides a concrete illustration of the framework. The conclusion that compliance is "omni-use" (incorporating both civilian and defense layers) is a direct result of the mapping exercise performed in Step 5.

**3. Constructive Criticism & Requirements for Scientific Soundness**

 To ensure the article is scientifically sound and fully reproducible, the authors must address the following points:

**A. Methodology & Replication (Crucial for "Partly" to "Yes")**

1. **Ontology Schema:** Provide a visual diagram of the **Regulatory-Compliance Ontology**. This should show the core classes, object properties, and data properties. Without this, the "automated" nature of the work remains a "black box" to the reader.
2. **Mapping Logic:** Step 4 describes "Mapping and Intersection." The authors should provide a more detailed table or logic flow showing exactly how a requirement from the AI Act (e.g., Transparency) was technically mapped to a NATO principle (e.g., Explainability).

**B. Data Availability & Reproducibility (Crucial for "Partly" to "Yes")**

1. **Repository Access:** The authors must provide a link to a public repository (e.g., GitHub or Zenodo) containing the .ttl (Turtle) or .owl files for the ontology and the Knowledge Graph used in the case study.
2. **Query Documentation:** While two SPARQL queries are provided, the paper should include the full set of queries used to generate the results in Table 1 to allow for verification of the automated compliance check.

**C. Technical Discussion (Enhancement)**

1. **Scalability:** The authors should briefly discuss the effort required to update the ontology when regulations change (e.g., an amendment to the AI Act). This is a known challenge in RegTech and addressing it would strengthen the "Academic Merit."
2. **Conflict Resolution:** If a defense requirement (NATO) contradicts a civilian requirement (GDPR) regarding data retention, how does the ontology handle this conflict? A paragraph on conflict-handling logic in semantic reasoning would be highly valuable.

**Final Recommendation**

 The manuscript is **technically strong and timely**. However, for it to meet the standards of full scientific reproducibility, the **ontological source files and visual schema must be made available**. Addressing the lack of a shared knowledge graph is the primary requirement for making the article scientifically sound.

Is the study design appropriate and does the work have academic merit?

Yes

Is the work clearly and accurately presented and does it cite the current literature?

Yes

If applicable, is the statistical analysis and its interpretation appropriate?

Not applicable

Are all the source data underlying the results available to ensure full reproducibility?

Partly

Are the conclusions drawn adequately supported by the results?

Yes

Are sufficient details of methods and analysis provided to allow replication by others?

Partly

Reviewer Expertise:

AI, Cybersecurity, Computer Vision, Machine Learning

I confirm that I have read this submission and believe that I have an appropriate level of expertise to confirm that it is of an acceptable scientific standard, however I have significant reservations, as outlined above.

# Response

**Published:** 2026-05-15

**Authors:** Giedre Sabaliauskaite

**Affiliations:** Mykolas Romeris University, Vilnius, Vilnius County, Lithuania

We thank the reviewer for their careful assessment of the manuscript and for identifying specific areas where the article can be strengthened. We acknowledge the points raised regarding methodology, reproducibility, ontology schema presentation, repository access, SPARQL query documentation, scalability, and conflict-handling logic. We will address these issues in the revised version of the manuscript by clarifying the ontology structure and mapping logic, improving the reproducibility materials, and expanding the technical discussion where appropriate. We appreciate the reviewer’s constructive guidance and will respond to each point in detail in the revised submission.
